# Cadherin-16 regulates acoustic sensory gating in zebrafish through endocrine signaling

**DOI:** 10.1371/journal.pbio.3003164

**Published:** 2025-05-02

**Authors:** Susannah S. Schloss, Zackary Q. Marshall, Nicholas J. Santistevan, Stefani Gjorcheska, Amanda Stenzel, Lindsey Barske, Jessica C. Nelson

**Affiliations:** 1 Department of Cell and Developmental Biology; University of Colorado Anschutz Medical Campus School of Medicine, Aurora, Colorado, United States of America; 2 Division of Human Genetics, Department of Pediatrics, Cincinnati Children’s Hospital Medical Center, University of Cincinnati College of Medicine, Cincinnati, Ohio, United States of America; University of Notre Dame, Center for Stem Cells and Regenerative Medicine, UNITED STATES OF AMERICA

## Abstract

Sensory thresholds enable animals to regulate their behavioral responses to environmental threats. Despite the importance of sensory thresholds for animal behavior and human health, we do not yet have a full appreciation of the underlying molecular-genetic and circuit mechanisms. The larval zebrafish acoustic startle response provides a powerful system to identify molecular mechanisms underlying establishment of sensory thresholds and plasticity of thresholds through mechanisms like habituation. Using this system, we identify Cadherin-16 as a previously undescribed regulator of sensory gating. We demonstrate that Cadherin-16 regulates sensory thresholds via an endocrine organ, the corpuscle of Stannius (CS), which is essential in zebrafish for regulating Ca^2+^ homeostasis. We further show that Cadherin-16 regulates whole-body calcium and ultimately behavior through the hormone Stanniocalcin 1l (Stc1l), and the IGF-regulatory metalloprotease, Papp-aa. Finally, we demonstrate the importance of the CS through ablation experiments that reveal its role in promoting normal acoustic sensory gating. Together, our results uncover a previously undescribed brain non-autonomous pathway for the regulation of behavior and underscore Ca^2+^ homeostasis as a critical process underlying sensory gating *in vivo.*

## Abstract 2

Sensory gating is a neuronal process that enables animals to distinguish between threatening and non-threatening stimuli. This study reveals a brain non-autonomous mechanism whereby cadherin-16-dependent changes in the endocrine organ corpuscle of Stannius regulate whole-body Ca2+ homeostasis and sensory gating in zebrafish.

## Introduction

Animals use sensory cues to evade threats in the environment. The acoustic startle response provides a crucial defensive mechanism, observed in species throughout the animal kingdom [1,2]. Although it is critical that animals be able to mount escape responses to threatening stimuli, they must also be able to distinguish between threatening and non-threatening stimuli. Sensory gating is a neural process that enables animals to make this distinction. Stimuli that do not meet a minimum stimulus intensity threshold do not elicit a response [3–5], and are ignored. Suprathreshold stimuli, conversely, are sufficient to elicit a behavioral response. In the case of the acoustic startle response in larval zebrafish, sensory thresholds are established during development and differ between animals, representing a form of behavioral individuality [6]. Moreover, thresholds established during development can be transiently modified through plasticity mechanisms like habituation [7,8]. In humans, a variety of neurological disorders, including schizophrenia, autism spectrum disorder, and migraine, are associated with differences in the ability to properly threshold or habituate to sensory stimuli [9,10]. Therefore, understanding the biological processes that regulate sensory thresholds may shed light on molecular mechanisms underlying disease.

Previous work has identified multiple molecular pathways that regulate sensory gating in larval zebrafish [3,4,11–15]. Many of these molecular regulators are expressed in or affect the activity of cells comprising the acoustic startle circuit, including the IGF-regulatory metalloprotease *pappaa* [11]*,* the voltage-gated K^+^ channel subunit *kcna1a* [14], the palmitoyltransferase *hip14* [14], the cytoskeletal regulator *cyfip2* [3]*,* and the Ca^2+^-sensing receptor *casr* [12,16]. Prior work has also probed key neurotransmitter signaling pathways that regulate acoustic startle response gating [7,8,17]. While this work has placed molecular mechanisms of behavior in the context of circuit function, most of the identified mechanisms function autonomously in the brain. How brain non-autonomous regulators of internal state, including whole-body homeostatic states might contribute is thus far largely unexplored.

In this study, we identify a novel brain non-autonomous mechanism key for promoting sensory gating. We find that Cadherin-16 (encoded by *cdh16*) functions in the pronephros-derived corpuscles of Stannius (CS) to regulate Ca^2+^ homeostasis and ultimately sensory thresholds in larval zebrafish. This system provides an ideal model for understanding how Ca^2+^ homeostasis regulates sensory thresholds. In particular, we find that Cdh16 regulates the expression of *stanniocalcin 1, like* (*stc1l*), a gene encoding the hormone Stanniocalcin 1l (Stc1l). Genes expressing Stanniocalcin hormones are present in vertebrates ranging from fish to humans where they regulate mineral homeostasis [18–20]. In mammals, Stc1 is expressed in multiple tissues including the kidney, while in the zebrafish, Stc1l is expressed by endocrine glands flanking the kidney called the corpuscles of Stannius [21,22]. Stc1l then functions to limit the proliferation and function of epithelial cells called ionocytes [23]. In particular, Stc1l limits the proliferation of a specific class of ionocytes, termed Na^+^/H^+^-ATPase-rich (NaR) cells, specialized to promote Ca^2+^ uptake from the environment [24]. Stc1l does this through the suppression of a metalloprotease, Papp-aa, expressed by NaR ionocytes [23]. Consequently, zebrafish *pappaa* loss-of-function mutants show reduced bone calcification [25]. PAPP-A is similarly crucial for Ca^2+^ homeostasis in mammals. Homozygous loss-of-function mutations in *PAPPA2* in humans are associated with growth deficits and reduced bone mineralization [26]. Interestingly, Papp-aa has also been identified as a key regulator of acoustic and visual behaviors in zebrafish [11].

Here we find that this *pappaa*-associated Ca^2+^-regulatory pathway functions in the context of sensory gating. Through genetic epistasis, we find that Cdh16 functions through Stc1l and ultimately Papp-aa to regulate whole-body Ca^2+^, which in turn broadly regulates behavioral thresholds, with opposite impacts on visually and acoustically evoked startle responses. Therefore, our results highlight a link between Papp-aa and Cdh16 function and underscore a crucial role for Ca^2+^ homeostasis in the regulation of sensory gating and behavior. Interestingly, human patient data also support a crucial role for Ca^2+^ homeostasis in the regulation of sensory gating: hypocalcemia in human patients is associated with seizures and psychotic symptoms, including auditory hallucinations [27,28].

## Results

### Cadherin-16 regulates acoustic startle response thresholds and habituation learning

At 5 days post-fertilization (dpf), larval zebrafish respond to high-intensity acoustic stimuli via a short-latency acoustic startle response, or short-latency C-bend (SLC) [2,29]. At this stage, zebrafish are also capable of distinguishing between high-intensity stimuli and low-intensity stimuli. They respond to high-intensity stimuli with fast responses and are able to ignore lower intensity stimuli or respond with long-latency C-bends [3,11,29]. Sensorimotor gating mechanisms, including the developmental establishment of acoustic startle response thresholds, enable animals to make distinctions between threatening and non-threatening acoustic stimuli [3]. Moreover, thresholds established during development can be transiently modified in 5 dpf larvae through plasticity mechanisms like habituation [7,8,11].

**Fig 1:**
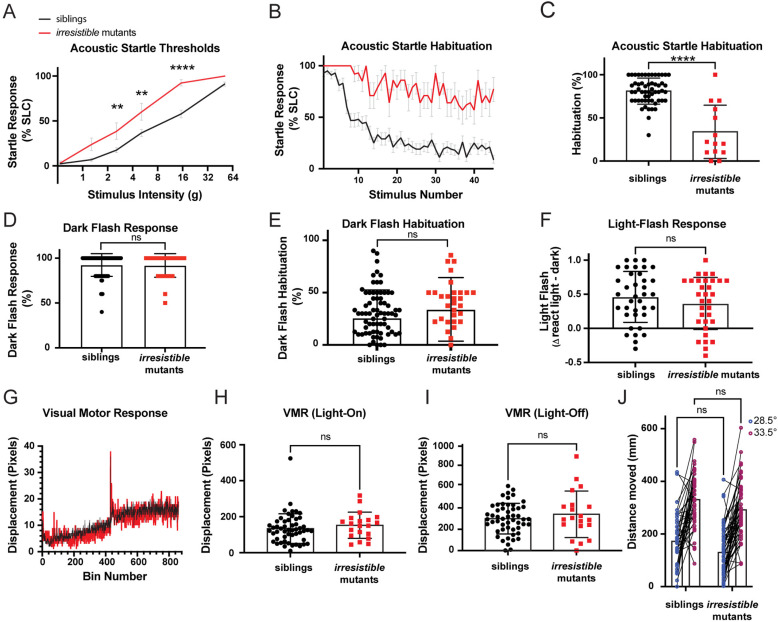
*Irresistible* mutations suppress habituation and cause hypersensitivity to acoustic stimuli. **(A)** *irresistible* mutants (*n* = 13) display heightened sensitivity to acoustic stimuli as compared to heterozygous and wild type (WT) siblings (*n* = 52). Error bars show SEM. Differences in startle sensitivity were calculated using a two-way ANOVA with a Šídák’s multiple comparisons test (**p < 0.01, ****p < 0.0001). **(B)** *irresistible* mutants (*n* = 14) fail to habituate to repeated acoustic stimuli when compared to siblings (*n* = 58), error bars show SEM. **(C)** *irresistible* mutants (*n* = 14) have lower habituation (****p < 0.0001, Mann–Whitney test) in relation to their siblings (*n* = 56). Error bars show SD. **(D)** *irresistible* mutants (*n* = 33) and siblings (*n* = 80) have no difference (*p* = 0.8615, Mann–Whitney test) in their response to dark flash stimuli. Error bars show SD. **(E)** *irresistible* mutants (*n* = 33) and siblings (*n* = 80) display no differences in habituation to dark flash stimuli (*p* = 0.0686, Mann–Whitney test). Error bars show SD. **(F)** *irresistible* mutants (*n* = 32) have no differences (*p* = 0.2983, unpaired *t* test) in ligh*t* flash reactivity as compared to their siblings (*n* = 37). Error bars show SD. **(G)** *irresistible* mutants (*n* = 20) display normal visual motor (VMR) behaviors relative to their siblings (*n* = 52). **(H)** *irresistible* mutants (*n* = 20) and siblings (*n* = 52) display no difference (*p* = 0.2471, Mann–Whitney test) in their responses to whole field illumination in VMR assay. **(I)** *irresistible* mutants (*n* = 20) and siblings (*n* = 52) do not show significantly different responses to whole field loss-of-illumination in VMR assay (*p* = 0.7223, Mann–Whitney test). Error bars show SD. **(J)** *irresistible* mutants (*n* = 54) and siblings (*n* = 42) have no significant differences in their movement at baseline temperature (*p* = 0.0877, two-way ANOVA with Šídák’s multiple comparisons test) and both respond to high temperature with increased locomotion (difference between mutants and siblings: *p* = 0.1231, two-way ANOVA with Šídák’s multiple comparisons test). The data underlying this figure can be found in S1 Data.

Through a forward genetic screen, a large collection of molecular regulators of sensorimotor gating were identified, including genes regulating (1) initial establishment of acoustic startle response thresholds, (2) plasticity of thresholds through habituation, and (3) the decision to perform a short-latency versus a long-latency C-bend [3,11–15]. *irresistible*^*p173*^ mutants were identified based on their inability to modulate response frequency through habituation and their hypersensitivity to low-level acoustic stimuli [11]. Specifically, a “sub-threshold” stimulus designed to elicit SLC responses only 5%–20% of the time elicited SLC responses more than 30% of the time in *irresistible* mutants. To understand further how acoustic startle thresholds are affected, we exposed *irresistible* mutants to a series of acoustic stimuli, ranging in intensity from 0.54*g* to 51.1*g* as previously described (see “Materials and methods”) [3,4]. *irresistible* mutants exhibit an increased sensitivity to acoustic stimuli, responding at higher rates than their siblings across multiple stimulus intensities, indicating deficits in sensory gating (Fig 1A). Next, we measured habituation by presenting animals with 40 high-intensity (51.1*g*) stimuli, each separated by a 3-s inter-stimulus interval (ISI). We found that *irresistible* mutants continue to respond at a high rate throughout the habituation assay, indicating deficits in the ability to dynamically tune acoustic startle response thresholds (Fig 1B and 1C).

Despite the dramatic impacts on their ability to threshold acoustic stimuli, *irresistible* mutants are adult-viable and fertile. To examine whether *irresistible* specifically regulates acoustic sensory gating, or has broader effects, we tested visual startle response rates (O-bend responses to whole-field loss of illumination or dark flash) [30] (Fig 1D), habituation to dark flash stimuli [7,31,32] (Fig 1E), light flash responses [30] (Fig 1F), visuomotor responses [33] (Fig 1G–1I), and ability to respond to thermal stimuli [34] (Fig 1J). We found no significant differences between *irresistible* mutants and their siblings, consistent with a specific deficit in developmental and acute regulation of acoustic thresholds in mutant larvae.

**Fig 2:**
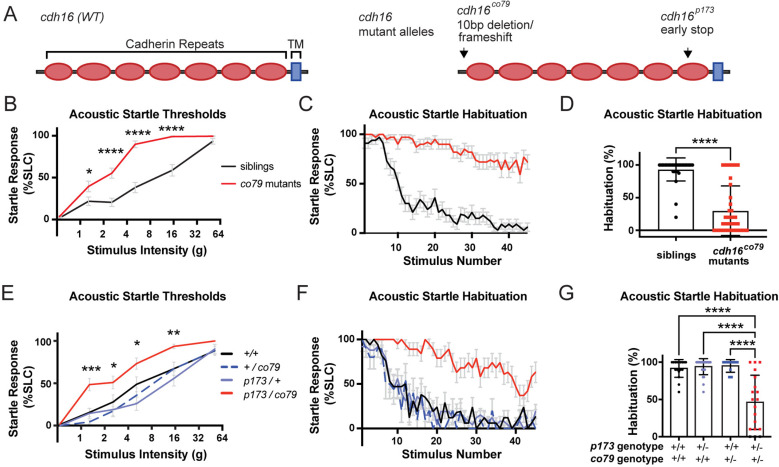
*Irresistible*^*p173*^ is an allele of the Cadherin-encoding gene *cdh16.* **(A)** Conceptual translation of *cdh16* and the predicted consequences of the *irresistible*^*p173*^*,* and *cdh16*^*co79*^ mutations. **(B)** *cdh16*^*co79*^ mutants (*n* = 39) have decreased thresholds to low intensity acoustic stimuli as compared to their siblings (*n* = 33) (*p = 0.0319, ****p < 0.0001, two-way ANOVA with Šídák’s multiple comparisons test). **(C)** *cdh16*^*co79*^ mutants (*n* = 39) continue responding to repeated acoustic stimuli while their siblings (*n* = 33) habituate. **(D)** *cdh16*^*co79*^ mutants (*n* = 39) have significantly impaired habituation (****p < 0.0001, Mann–Whitney test) compared to siblings (*n* = 32). **(E)** *cdh16*^*p173*^/*cdh16*^*co79*^ transheterozygotes (*n* = 22) have increased sensitivity to acoustic stimuli when compared to *cdh16*^*p173*^ heterozygotes (*n* = 17), *cdh16*^*co79*^ heterozygotes (*n* = 14), and wild types (*n* = 16). A two-way ANOVA with Tukey’s multiple comparisons test was used to calculate the difference in SLC% between all groups. Differences between *cdh16*^*p173/co79*^ versus WT (+/+) are represented with *p* values on the plot: ***p = 0.0005, for the difference between WT and transheterozygotes, *p < 0.03, **p = 0.0086. **(F)** *cdh16*^*p173*^/*cdh16*^*co79*^ transheterozygotes (*n* = 19) fail to habituate to high intensity acoustic stimuli while wild type (*n* = 17), *cdh16*^*co79*^ heterozygotes (*n* = 10), and *cdh16*^*p173*^ heterozygotes (*n* = 26) habituate normally. **(G)** *cdh16*^*p173*^/*cdh16*^*co79*^ transheterozygotes (*n* = 19) have significantly lower habituation percentages *p* < 0.0001 compared to wild types (*n* = 17), *cdh16*^*co79*^ heterozygotes (*n* = 10), and *cdh16*^*p173*^ heterozygotes (*n* = 25). Differences in habituation between groups were calculated using a two-way ANOVA with Tukey’s multiple comparisons test. Error bars in **B, C, E**, and **F** indicate SEM. Error bars in **D** and **G** indicate SD. The data underlying this figure can be found in S1 Data.

To map the genetic locus responsible for the *irresistible* phenotype, we conducted whole-genome sequencing followed by homozygosity mapping as previously described [11]. This uncovered a premature stop codon (Y657*) in the *cdh16* gene, encoding the calcium-dependent cell-adhesion protein, Cadherin-16. Like other members of the 7D family of cadherins, Cadherin-16 has 7 extracellular cadherin domains, a transmembrane domain (TM), and a short intracellular domain [35,36]. Y657* results in a termination codon after the sixth Cadherin domain, prior to the TM domain (Fig 2A). To determine whether the premature stop codon in the *cdh16* locus is causal for the acoustic hypersensitivity and habituation phenotypes, we used CRISPR-Cas9 genome editing to generate an independent loss-of-function allele, *co79*, in *cdh16* in wild type animals. *co79* results in a 10 bp deletion in exon 2, resulting in a frameshift and premature stop codon (F38del[SPSCQISL*]FSX8) (Fig 2A). Like *p173*, animals homozygous for the *co79* mutant allele are hypersensitive to acoustic stimuli and fail to habituate (Fig 2B–2D). Conversely, habituation and startle sensitivity assays demonstrated that animals heterozygous for either *co79* or *p173* are indistinguishable from their wild type siblings on these measures (Fig 2E–2G). Finally, through complementation testing, we determined that larvae carrying a combination of both mutant alleles (*cdh16*^*p173/co79*^) fail to habituate and exhibit hypersensitivity to low-intensity acoustic stimuli (Fig 2E–2G). Together, these data demonstrate that Cadherin-16 regulates the establishment and dynamic tuning of acoustic startle thresholds through habituation.

### Cadherin-16 expression is sufficient after the development of the acoustic startle circuit to restore acoustic startle thresholds and habituation

**Fig 3:**
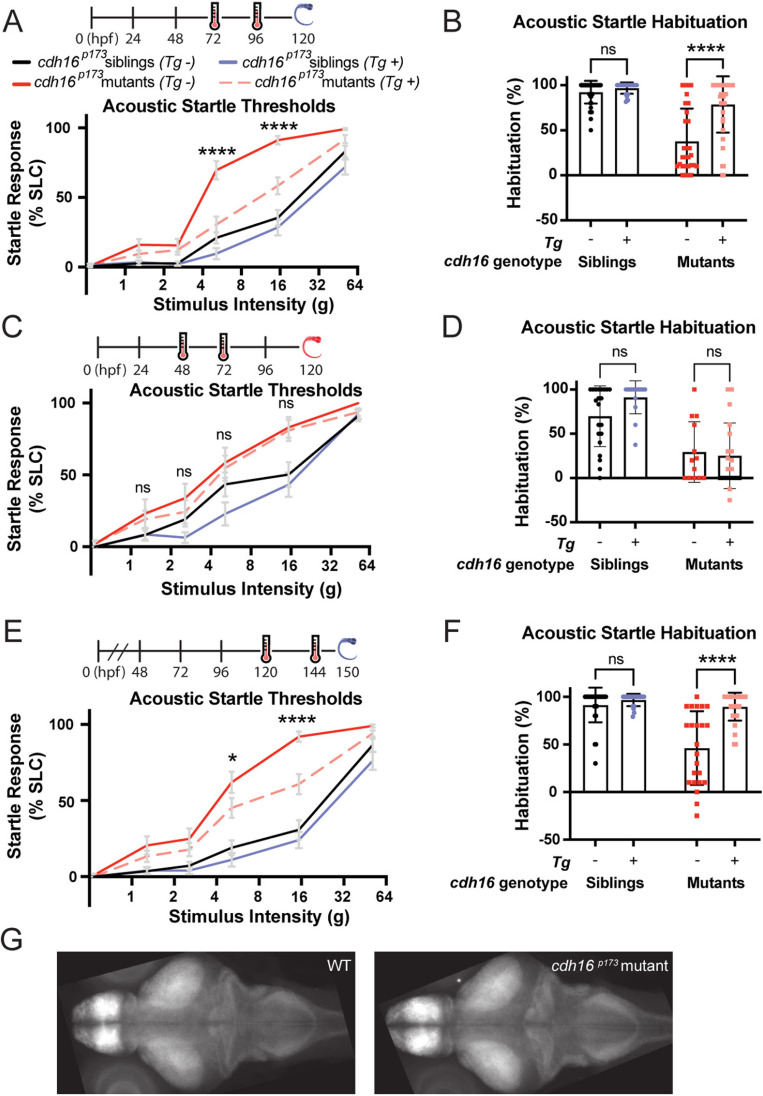
Ubiquitous expression of *cdh16* after circuit development restores habituation and acoustic sensitivity. **(A)** *hsp70p:cdh16-p2a-mKate* expression was induced at 72 and 96 hpf (hours post-fertilization) via heat-shock. Behavior testing and analysis performed at 120 hpf. Induction of *cdh16* expression in *cdh16*^*p173*^ mutants (*n* = 38) results in significantly lower startle sensitivity compared to *cdh16*^*p173*^ mutants that are heat-shocked but do not carry the transgene (*n* = 25). ****p < 0.0001. **(B)** Heat-shock as in **A** has no effect on habituation (*p* = 0.7204) of siblings (*n* = 41 with the transgene versus *n* = 26 without). In contrast, heat-shock induction of *cdh16* expression significantly restores habituation (*p* < 0.0001) in *cdh16*^*p173*^ mutants carrying the transgene (*n* = 38) in comparison to transgene negative mutants (*n* = 29). **(C)** *hsp70p:cdh16-p2a-mKate* expression was induced at 48 and 72 hpf via heat-shock. Behavior testing and analysis performed at 120 hpf. Acoustic startle sensitivity is not significantly restored in *cdh16*^*p173*^ mutants carrying the heat-shock transgene (*n* = 19) when compared to mutants with no transgene (*n* = 13). These data are consistent with a requirement for maintenance of *cdh16* expression during behavior (*p* > 0.7 for all stimulus intensities). **(D)** Heat-shock as in **C** has no effect on habituation (*p* = 0.1073) in siblings expressing the transgene (*n* = 15) in relation to siblings not expressing the transgene (*n* = 21). Similarly, the difference in acoustic startle habituation in transgene-expressing mutants (*n* = 19) and mutants not expressing the transgene (*n* = 13) is not significant (*p* = 0.9199). **(E)** *hsp70p:cdh16-p2a-mKate* expression was induced at 120 and 144 hpf (after the acoustic startle circuit is functional) via heat-shock. Behavior testing and analysis performed at 150 hpf. Hypersensitivity is rescued in *cdh16*^*p173*^ mutants (*n* = 27) carrying the transgene as compared to mutants lacking the transgene (*n* = 20). (*p = 0.0380, ****p < 0.0001). **(F)** Heat-shock as in **E** has no effect on acoustic startle habituation (*p* = 0.6607) in siblings carrying the transgene (*n* = 21) compared to siblings lacking the transgene (*n* = 28). Conversely, *cdh16* expression restores habituation to acoustic stimuli (*p* < 0.0001) in mutants carrying the transgene (*n* = 31) as compared to mutants without the transgene (*n* = 23). For **A, C, and E,** error bars indicate SEM. For **B, D, and F**, error bars indicate SD. **(G)** Representative whole-brain stacks for WT (*n* = 16) (left) and *cdh16*^*p173*^ mutants (*n* = 13) (right), showing a lack of brain volume changes at 6 dpf. The data underlying this figure can be found in S1 Data.

The neuronal circuits required for the performance of the acoustic startle response are in place by 4 dpf. By this stage, animals reliably perform acoustic startle responses to high-intensity stimuli and exhibit robust habituation learning [2,37]. Cadherin proteins regulate many developmental processes throughout the body, including the assembly of neuronal circuits [38]. Therefore, we wondered whether *cdh16* is required for the assembly of the acoustic startle circuit, or whether it might be required for the maintenance, function, or maturation of the acoustic startle circuit. To test this, we generated a transgene expressing *cdh16* under the control of the *hsp70* heat-shock activated promoter. We found that ubiquitous, heat-shock induced expression of *cdh16* at 3 and 4 dpf rescued acoustic startle thresholds and habituation at 5 dpf, consistent with a role for Cdh16 during development. The same manipulation had no significant effect in sibling animals overexpressing *cdh16* (Fig 3A and 3B). However, expression of *cdh16* at 2 and 3 dpf did not restore normal behavior measured at 5 dpf, suggesting that maintenance of Cadherin-16 expression at the time of behavior testing is required for the regulation of sensory-evoked behaviors (Fig 3C and 3D). Moreover, we determined that heat-shock induced expression of *cdh16* at 5 and 6 dpf rescued acoustic startle thresholds and habituation measured six hours after heat shock on day 6, consistent with a role for Cdh16 in the regulation of sensory processing after the establishment of the acoustic startle circuit (Fig 3E and 3F). To broadly examine how *cdh16* might impact neuronal development, we performed whole-brain morphometric analyses in *cdh16* mutants versus siblings across 294 molecularly-defined brain regions [39,40]. This unbiased approach for assessing brain development revealed no substantial changes in size across these regions (Figs 3G, S1). Although this approach is designed to detect substantial changes in region-by-region volume, and likely would not resolve finer changes, these findings are consistent with our heat-shock rescue data and underscore a role for Cdh16 in regulating nervous system function rather than early nervous system development.

### *cdh16* is expressed in the corpuscles of Stannius

**Fig 4:**
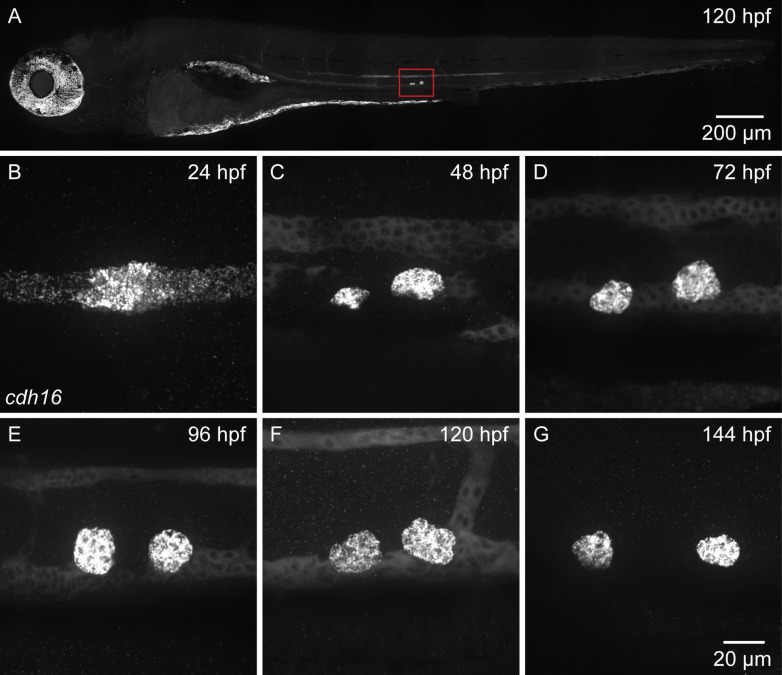
*cdh16* is expressed in the corpuscles of Stannius (CS) during embryonic and larval development. **(A–G)** Whole-mount *in situ* hybridization chain reaction (HCR) using probes against sequences contained within the *cdh16*^*co120*^ large deletion **(A)**, or full *cdh16* probe set **(B–G)***.* Maximum projections of confocal stacks show the whole larval zebrafish **(A),** pronephros **(B),** and corpuscles **(C–G)**. **(A)** At 120 hpf, *cdh16* is expressed in the corpuscles of Stannius (red box). Background signal is observed elsewhere, including in the eyes and head. **(B)** *cdh16* puncta are enriched in distal pronephros where the CS will be extruded. **(C–F)** *cdh16* signal is present in the CS and kidney from 48 hpf to 120 hpf. **(G)** By 144 hpf *cdh16* signal is present in the CS but is no longer detectable in the kidney. Shown are representative images, *n* = 5 larvae were imaged for whole-body and per time point.

In mammals, *cdh16* is primarily expressed in the kidney and the thyroid [41,42]. In zebrafish, while *cdh16* expression in the brain has been documented at 10 days post-fertilization [43], others have found that at earlier stages *cdh16* is primarily expressed in the developing pronephros or embryonic kidney [44]. Around 2 days post-fertilization, *cdh16* expression becomes largely restricted to an endocrine organ called the corpuscle of Stannius (CS), which is extruded from the pronephros and secretes Stc1l, a Ca^2+^-regulatory hormone [44]. Given our finding that *cdh16* is required for sensory gating after 2 dpf, we wondered whether *cdh16* expression might persist in the CS beyond this early developmental time point. To address this question, we used *in situ* hybridization chain reaction (HCR), examining *cdh16* expression in whole-mount embryos and larvae from 24 h post-fertilization (hpf) through 144 hpf (Fig 4A–4G). At 5 dpf, we observed strong *cdh16* expression in the CS. Consistent with earlier reports of *cdh16* expression [44], we also observed weak signal in other tissues, including in the head (Fig 4A). To distinguish between true *cdh16* expression and non-specific probe binding, we generated a large deletion in the *cdh16* locus, *cdh16*^*co120*^. We then designed an HCR probe set, in which all probes bind to target sequences contained within the large deletion (S2A Fig). Finally, we repeated our HCR experiment in *cdh16*^*co120*^ mutant animals. In mutant larvae carrying the large deletion, we found that signal in the CS was lost, indicating that this signal reports true *cdh16* expression. Conversely, weak signal observed in other tissues, including the head, was maintained, consistent with this signal arising due to non-specific probe binding (S2B Fig). These findings are consistent with Daniocell data that indicate larval expression of *cdh16* in the CS, but not in neural or glial cell types [45,46]. Indeed, at all time points, we found that *cdh16* was strongly expressed either in the pronephros (24 hpf, Fig 4B) or the CS (48–144 hpf) (Fig 4C–4G), and we have no evidence to support *cdh16* expression in the brain at 5 dpf. Based on these data, we predicted that Cdh16 is required outside the brain to regulate acoustic startle thresholds.

### Cadherin-16 promotes the function of PAPP-AA through the regulation of the hormone Stanniocalcin 1l

**Fig 5:**
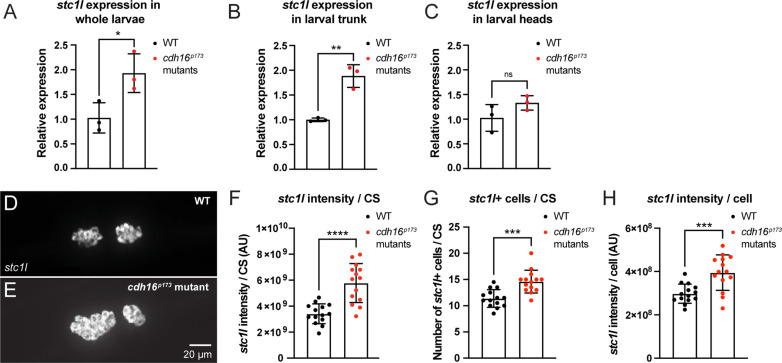
Cadherin-16 suppresses *stc1l* expression in the CS. **(A–C)** RT-qPCR analysis of *stc1l* expression in *cdh16*^*p173*^ mutants. **(A)** Expression of *stc1l* is significantly increased in *cdh16* mutants compared to WT. *n* = 3 biological replicates per condition, *n* = 7 larvae per biological replicate, *p = 0.03 unpaired *t* test. Error bars represent SD. **(B–C)** The increase in *stc1l* expression in *cdh16* mutants is observed specifically in trunk tissue, which includes the distal pronephros and CS **(B)** *n* = 3 biological replicates per condition, *n* = 10 larvae per biological replicate, **p = 0.003, unpaired *t* test, and not the head **(C)** *n* = 3 biological replicates per condition, *n* = 10 larvae per biological replicate, ns indicates *p* = 0.16, unpaired *t* test. Error bars represent SD. **(D-H)** *stc1l in situ* HCR in the CS of *cdh16*^*p173*^ mutants. **(D–E)** Comparison of the corpuscles of Stannius (CS) between *cdh16* mutants (bottom) and WT siblings (top). **(F–H)** *cdh16* mutants (*n* = 15) have increased *stc1l* expression per CS (****p < 0.0001 unpaired *t* test) **(F)**, increased *stc1l*-positive cells per CS (***p = 0.0002 unpaired *t* test) **(G)**, and increased *stc1l* expression per CS cell (***p = 0.0006), compared to WT (*n* = 15) **(H)**. Error bars represent SD. The data underlying this figure can be found in S1 Data.

Morpholino knockdown of *cdh16* in embryonic zebrafish leads to a dramatic increase in the expression of *stc1l* [44]. In zebrafish and mammals, Stc1 inhibits the metalloprotease PAPP-AA [23,25,47,48], which is a known regulator of acoustic startle sensitivity and habituation in larval zebrafish [11]. *pappaa* mutants largely phenocopy *cdh16* with one exception: *pappaa* mutants are not responsive to dark-flash, or whole-field loss of illumination [11,49]. We hypothesized that excessive *stc1l* expression in *cdh16* mutants inhibits *pappaa,* precluding appropriate acoustic startle thresholding and plasticity of thresholds through habituation. To test our hypothesis, we set out to confirm that *cdh16* mutants, like *cdh16* morphants, show increased expression of *stc1l*. Using RT-qPCR, we found that as in *cdh16* morphants, *stc1l* expression was strongly increased in *cdh16* loss-of-function mutants (Fig 5A). Next, we wondered whether loss of *cdh16* might lead to a change in *stc1l* expression in the brain. To test this, we dissected 5 dpf larval zebrafish, separating the trunk and the head, and performed RT-qPCR in each tissue independently in mutants and siblings. We found that while *stc1l* was strongly upregulated in the trunk (which contains the CS) (Fig 5B), there was no change in the head (Fig 5C), consistent with a CS-specific role of Cdh16 in regulating *stc1l* expression. Finally, we wondered how Cdh16 might suppress *stc1l* expression. Recent work shows that mutations in *sox10* increase the number of *stc1l*-positive cells in the CS, consistent with a possible role in regulating the proliferation or survival of CS cells [50]. To test whether a similar mechanism might underly the role of Cdh16 in suppressing *stc1l* expression, we performed HCR for *stc1l* in *cdh16*^*p173*^ mutant and sibling animals (Fig 5D and 5E), quantifying the *stc1l* signal per CS as well as the number of cells comprising each CS. Consistent with our RT-qPCR data, we found that *stc1l* signal per CS was increased in *cdh16* mutants relative to their siblings (Fig 5F). Moreover, consistent with a role for Cdh16 in regulating the proliferation or survival of CS cells, we found that the total number of cells comprising each CS was increased in *cdh16* mutant animals relative to their siblings (Fig 5G). Next, we quantified *stc1l* signal per cell and found that this was also increased in *cdh16* mutants (Fig 5H), consistent with a role for Cdh16 in regulating both *stc1l* expression and proliferation or survival of *stc1l-*expressing cells. Finally, we wondered whether loss of *cdh16* might affect the development of the Mauthner cell, a reticulospinal neuron that receives acoustic inputs from the eighth nerve, and sends outputs to motor neurons along the spinal cord, driving the SLC behaviors that are hypersensitive in *cdh16* mutant animals. We measured Mauthner soma length (S3A Fig), lateral dendrite length (S3B Fig), and total Mauthner length from the tip of the lateral dendrite to the axon initial segment (S3C Fig). None of these three measures were significantly different between mutant and WT animals (S3D and S3E Fig). Together, these data indicate that Cdh16 functions in the CS to suppress *stc1l* expression in part by suppressing proliferation or maintenance of cells comprising this endocrine organ.

**Fig 6:**
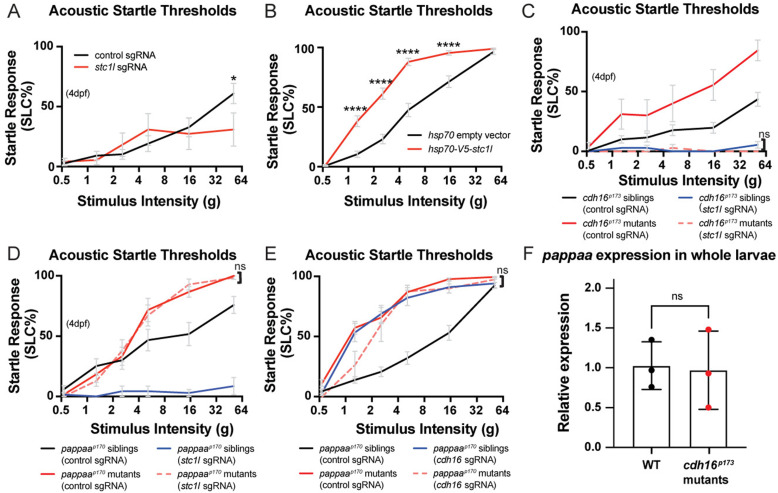
Cadherin-16 promotes startle thresholds by limiting Stanniocalcin 1l expression and promoting Papp-aa function. **(A)** *stc1l* crispants (*n* = 18) have a decreased response to acoustic stimuli compared to control guide-injected larvae (*n* = 18) *p = 0.0475, two-way ANOVA with Šídák’s multiple comparisons test. Error bars represent SEM. **(B)** Heat-shock overexpression of V5-tagged *stc1l* at 4 dpf causes increased sensitivity to acoustic stimuli at 5 dpf (*n* = 54) compared to overexpression of empty heat-shock vector (*n* = 53) (****p < 0.0001, two-way ANOVA with Šídák’s multiple comparisons test). Error bars represent SEM. **(C)** Genetic epistasis to examine the relationship between *stc1l* and *cdh16* in the context of acoustic startle thresholds. *stc1l* mutations suppress the *cdh16* mutant phenotype. *cdh16* mutants injected with *stc1l* guides (*n* = 10) are not more responsive than siblings injected with *stc1l* guides alone (*n* = 44) *p* > 0.9 for all stimulus intensities, two-way ANOVA with Tukey’s multiple comparisons test. Error bars represent SEM. **(D)** *pappaa* mutations suppress the *stc1l* crispant phenotype. *stc1l* guide-injected *pappaa* mutant larvae (*n* = 14) are no more hyposensitive than control guide injected *pappaa* mutants (*n* = 12) *p* > 0.9827 for all stimulus intensities, two-way ANOVA with Tukey’s multiple comparisons test. Error bars represent SEM. **(E)** Loss-of-function mutations in *cdh16* and *pappaa* do not cause additive hypersensitivity phenotypes. *pappaa* mutants injected with *cdh16* guides (*n* = 8) are no more hypersensitive than control guide injected *pappaa* mutants (*n* = 18), *p* > 0.8 at all intensities except for 1.3*g*, where *p* = 0.0389, and control-guide injected are more sensitive than *cdh16* guide-injected *pappaa* mutants, two-way ANOVA with Tukey’s multiple comparisons test. **(F)** RT-qPCR analysis of *pappaa* mRNA levels. *pappaa* expression is not altered in *cdh16*^*p173*^ mutants as compared to their WT siblings (*n* = 3 biological replicates per condition, *n* = 7 larvae per biological replicate, *p* = 0.87, unpaired *t* test). Error bars represent SD. The data underlying this figure can be found in S1 Data.

Based on these findings, we predicted that since hypersensitive *cdh16* mutants overexpress *stc1l*, *stc1l* loss-of-function would lead to hyposensitivity to acoustic stimuli. To test this, we performed *F*_0_ CRISPR mutagenesis experiments, injecting wild type embryos at the 1-cell stage with Cas9 together with either 3 control guides [51] or together with 3 guides that we designed against *stc1l*. We found that loss of function in *stc1l* leads to severe pericardial edema, which becomes apparent by 5 dpf as previously described [23]. Therefore, we tested behavior in larvae injected with *stc1l* guides (*stc1l* crispants) at 4 dpf, before severe pericardial edema develops. At 4 dpf, wild type zebrafish larvae are less responsive to acoustic stimuli, but as predicted, we found that *stc1l* crispants were even less responsive to acoustic stimuli than their control guide injected siblings (Fig 6A). Next, to test our hypothesis that the *cdh16* mutant phenotype arises due to overexpression of Stc1l, we injected a construct expressing *V5-stc1l* under the control of a heat-shock promoter to transiently overexpress Stc1l in wild type embryos. We then heat-shocked the injected embryos (as well as embryos injected with a construct containing the heat-shock promoter alone) at 4 dpf and tested their behavior the next day at 5 dpf. Consistent with our hypothesis that hypersensitivity is a result of overexpressed Stc1l, animals transiently overexpressing Stc1l were hypersensitive to acoustic stimuli relative to control-injected siblings (Fig 6B).

Next, we set out to test whether *stc1l* overexpression in *cdh16* mutants is the cause of the hypersensitivity phenotype. For this, we performed the same CRISPR-Cas9 *F*_0_ mutagenesis in *cdh16* mutants and siblings. Consistent with our model, *stc1l* loss-of-function in *cdh16* mutants resulted in hypo-responsiveness to acoustic stimuli (Fig 6C).

Previous work shows that Stc1l limits Ca^2+^ uptake by inhibiting Papp-aa [23]*.* Therefore, we predicted that *pappaa* loss-of-function would suppress the hyposensitive phenotype observed in *stc1l* crispants, and that loss-of-function of both genes would resemble single mutants for *pappaa*. Indeed, we found that *pappaa* mutants injected with *stc1l* guides were hypersensitive, showing no difference relative to control-guide injected *pappaa* mutants (Fig 6D). If the function of *cdh16* is to release *pappaa* from inhibition by inhibiting *stc1l,* then animals carrying loss-of-function mutations in both *cdh16* and *pappaa* should be no more hypersensitive to acoustic stimuli than single mutants for either gene. Indeed, our crispant experiments are consistent with this model, as *pappaa* mutants injected with guides against *cdh16* were no more hypersensitive than *pappaa* mutants injected with control guides (Fig 6E). Finally, we set out to understand whether loss of *cdh16* affects expression of *pappaa.* Stc can both down-regulate the expression of *pappaa* [23] and separately can inhibit its enzymatic function [47,48]. To address this question, we conducted RT-qPCR experiments to assess whole-body *pappaa* expression in *cdh16*^*p173*^ mutants relative to WT. We found that *pappaa* RNA expression levels were not changed in our *cdh16* mutants relative to WT (Fig 6F). Together, our data support a model in which Cdh16 suppresses *stc1l* and promotes *pappaa* function rather than directly regulating its expression, though future experiments are needed to carefully assess the enzymatic function of *pappaa* in *cdh16* mutant animals.

### The corpuscles of Stannius and Ca^2+^ homeostasis are crucial regulators of acoustic sensory thresholds

**Fig 7:**
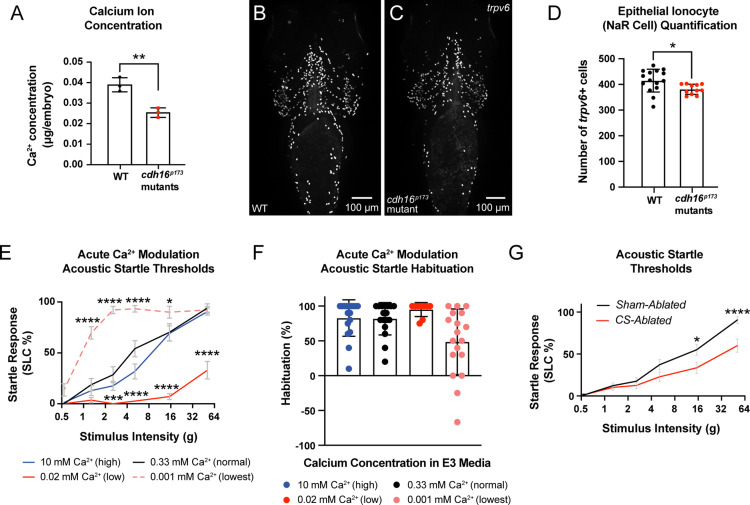
The corpuscles of Stannius (CS) and Ca^2^ ^**+**^ **homeostasis are important regulators of behavioral thresholds.** **(A)** *cdh16* mutants have decreased whole-body Ca^2+^ compared to WT (*n* = 3 biological replicates per condition, **p = 0.0048, unpaired *t* test). Error bars represent SD. **(B–C)** Epithelial ionocytes involved in Ca^2+^ uptake (NaR cells) visualized via whole mount *in situ* HCR for *trpv6* in WT **(B)** and *cdh16*^*p173*^ mutants **(C)**. **(D)** *cdh16* mutants (*n* = 11) have fewer ionocytes compared to WT (*n* = 15) *p = 0.0278, unpaired *t* test. Error bars represent SD. **(E–F)** Acute (four hour) exposure to media with altered Ca^2+^ concentration alters sensory thresholds and habituation to acoustic stimuli. **(E)** Larvae exposed to the lowest concentration of Ca^2+^ (0.001 mM Ca^2+^) four hours before behavior testing have increased sensitivity to acoustic stimuli (*n* = 18) compared to larvae exposed to normal levels of Ca^2+^ (0.33 mM) (*n* = 17); ****p < 0.0001, *p = 0.02. Larvae exposed to an intermediate-low level of Ca^2+^ (0.02 mM, *n* = 17) conversely, have reduced responses to acoustic stimuli relative to normal Ca^2+^ (0.33 mM) (*n* = 17) ***p = 0.0004, ****p < 0.0001, two-way ANOVA with Dunnett’s multiple comparison’s test. Error bars represent SEM. **(F)** Larvae in the lowest concentration of Ca^2+^ trended towards a failure to habituate to acoustic stimuli (*n* = 18) relative to larvae exposed to normal levels of Ca^2+^ (0.33 mM, *n* = 17) *p* = 0.0653, Kruskal–Wallis test with Dunn’s multiple comparisons test. Error bars represent SD. **(G)** Laser-ablation of the Ca^2+^-regulatory corpuscles of Stannius (CS) causes decreased sensitivity to acoustic stimuli (*n* = 20), compared to sham ablated siblings (*n* = 20) *p = 0.012, ****p < 0.0001, two-way ANOVA with Šídák’s multiple comparisons test. Error bars indicate SEM. The data underlying this figure can be found in S1 Data.

Thus far, our data are consistent with a model in which Cdh16 and Papp-aa regulate Ca^2+^ homeostasis to promote acoustic startle thresholds and habituation. Importantly, in addition to its expression in Ca^2+^-regulatory ionocytes, *pappaa* is expressed in cells surrounding neuromasts, as well as in the retina and brain, including in the acoustic startle circuit [11,25,49,52,53]. However, it is not yet known whether *pappaa* expression in the brain or potentially in the ionocytes regulates sensory thresholds. First, to test whether *cdh16* mutants are hypocalcemic, we performed a colorimetric assay for whole-body Ca^2+^ content. Consistent with a model in which loss of *cdh16* leads to excessive *stc1l*, which downregulates *pappaa* and ionocyte proliferation and function to ultimately impair Ca^2+^ uptake, we found that *cdh16* mutants are hypocalcemic relative to their siblings (Fig 7A). Next, we wondered whether *cdh16* mutants might be hypocalcemic as a result of impaired ionocyte proliferation. To assess this, we used HCR probes against *trpv6* to label NaR ionocytes in the ventral epithelium. Using the *trpv6* signal to count individual ionocytes in mutants versus siblings, we found that mutant animals had a subtle, but significant reduction in NaR ionocyte number (Fig 7B–7D). These data are consistent with loss of *cdh16* resulting in overexpression of *stc1l*, hyper-suppression of Papp-aa function, and ultimately resulting in reduced ionocyte proliferation, reduced Ca^2+^ uptake, and impaired gating. Supporting the idea that reduced Ca^2+^ might cause hypersensitivity to acoustic stimuli, prior work has demonstrated that zebrafish raised in high-Ca^2+^ media are hyposensitive to acoustic stimuli [54]. To test whether low Ca^2+^ media would result in acoustic hypersensitivity, we exposed larvae to low-Ca^2+^ media (0.001 mM) for 4 h. We found that this short-term treatment resulted in acoustic hypersensitivity (Fig 7E) and animals exposed to this treatment trended towards a failure to habituate (Fig 7F). We note that short-term exposure to 0.02 mM Ca^2+^ surprisingly caused the opposite phenotype: animals were hyposensitive and trended toward improved habituation. Finally, to test how short-term exposure to media with altered Ca^2+^ concentration affects whole-body Ca^2+^, we again used our colorimetric assay (S4A Fig). We found that while high Ca^2+^ (10 mM) significantly elevated whole-body Ca^2+^, exposure to low Ca^2+^ trended toward a lower level of whole-body Ca^2+^, but was not significant (*p* = 0.093).

We additionally examined visually evoked behaviors (S4B–S4D Fig). Like *pappaa* mutants*,* animals exposed to low Ca^2+^ media (0.001 mM) show reduced responsiveness to dark-flash stimuli, consistent with low Ca^2+^ in *pappaa* mutants as an important driver of both phenotypes. These data highlight that low Ca^2+^ and loss of *pappaa* both cause reduced escape responses to whole-field loss of illumination and increased responsiveness to acoustic stimuli.

Finally, our data suggest that *cdh16* regulates sensory thresholds through its function in the CS. To test this, we used a 532 nm pulse laser to ablate the CS in wild type animals expressing *her6:mCherry* [55], a transgene that labels the CS at 3–4 dpf. Based on the overexpression of *stc1l* in the CS of *cdh16* mutants, and the suppression of hypersensitivity in *cdh16*^*p173*^*; stc1l* crispants, we predicted that ablation of the CS would result in hyposensitivity similar to that observed in *stc1l* crispant animals*.* Importantly, CS-ablated animals largely did not display pericardial edema at 5 dpf (S4E and S4F Fig). Those with pericardial edema were excluded from analysis. Consistent with a function for *cdh16* in the CS, we found that compared to their sham-ablated counterparts, CS-abated wild type animals were hyposensitive to acoustic stimuli (Figs 7G and S4G–S4J).

## Discussion

**Fig 8:**
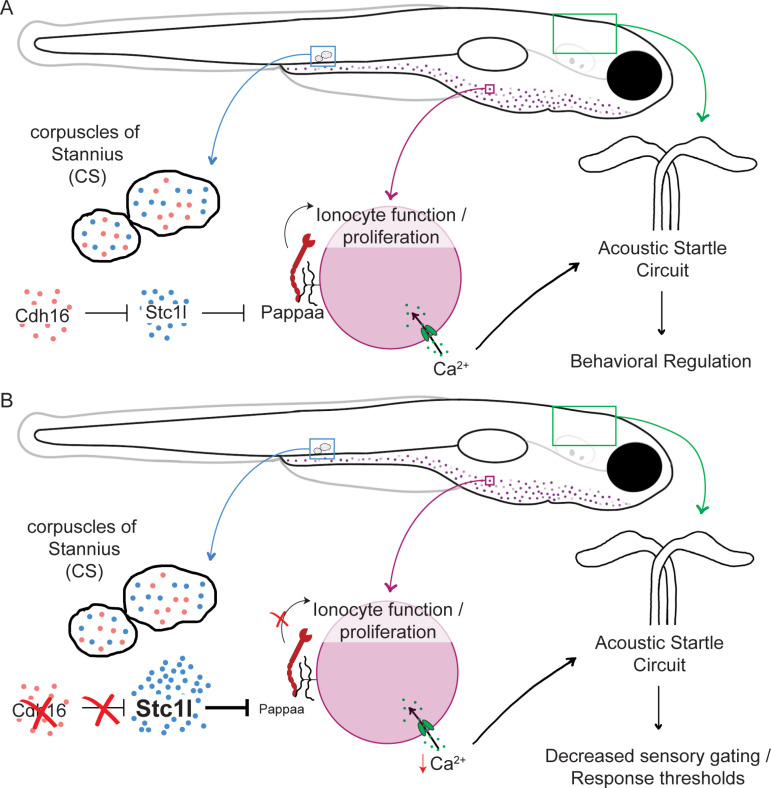
Proposed model. **(A)** In wild type animals, Cdh16 suppresses *stc1l* expression in the corpuscles of Stannius. This limits the ability of Stc1l to suppress the function of Papp-aa, allowing for some proliferation and function of ionocytes. As a result, Ca^2+^ is taken up from the environment and normal acoustic startle thresholds are maintained. **(B)** In *cdh16* mutant animals, suppression of *stc1l* expression is relieved and *stc1l* is overexpressed. This results in hyper-inhibition of Papp-aa. As a result, Ca^2+^ uptake is severely limited, animals are hypocalcemic, and acoustic response thresholds are lowered.

Taken together, our results highlight the corpuscles of Stannius as brain non-autonomous endocrine regulators of sensory thresholds. Moreover, our results identify Cadherin-16 as an important regulator of endocrine function and highlight Ca^2+^ homeostasis as critical for sensory gating *in vivo*. Based on our data, we favor a model in which without *cdh16*, cells of the CS over-proliferate, producing excess Stc1l. Over-expressed Stc1l then hyper-suppresses Papp-aa function at least in part at the level of NaR ionocytes, where Papp-aa would ordinarily support their proliferation. In *cdh16* mutants, however, ionocytes proliferate less, and insufficient Ca^2+^ is taken up from the environment. The ultimate consequence is that zebrafish larvae are hypocalcemic, leading to hypersensitivity to acoustic stimuli and in the case of *pappaa* loss-of-function, insufficient responding to whole-field loss of illumination (dark flash response) (Fig 8A and 8B).

We find that Cdh16 regulates Stc1l in two different ways. First, we find that in *cdh16* mutants, there are more *stc1l*+ cells comprising each CS, suggesting that Cdh16 plays a role in regulating cell proliferation or survival. Nonetheless, we additionally find that *stc1l* expression per CS cell is higher in *cdh16* mutants, consistent with a model in which Cdh16 both suppresses cell proliferation and separately suppresses *stc1l* expression within those cells. The molecular mechanisms underlying these roles for Cadherin-16 are not yet known. Cadherin-16 is an atypical cadherin within the 7-domain family of cadherins and characterized by a short intracellular domain lacking binding sites for catenins. Therefore, although Cadherin-16 can function as an adhesion protein [35], the intracellular mechanisms underlying Cadherin-16 regulation of cell proliferation or survival and *stc1l* expression are not yet known. These questions are relevant to our understanding of sensory gating and the development of the CS, but also for cancer biology, as *cdh16* is downregulated in thyroid carcinomas [56] and limits thyroid carcinoma cell proliferation [57].

We find that Cdh16 and Stc1l function to regulate Papp-aa. Papp-aa fully suppresses the *stc1l* loss-of-function phenotype, and loss of both *pappaa* and *cdh16* does not result in additive behavioral deficits. Papp-aa is expressed in ionocytes, cells surrounding the lateral line neuromasts, and in the retina [49,52,53]. We now present data to support that it regulates behavior in part via regulating the proliferation of NaR ionocytes in the skin. Specifically, we show that these cells are reduced in number in *cdh16* mutant animals, providing a systems-level mechanism to explain how loss of *cdh16* leads to lower Ca^2+^. Nonetheless, we do not yet know whether *pappaa* might also regulate the invasion of ionocyte precursors into neuromasts and subsequent differentiation of the recently described neuromast-resident ionocytes [58,59]. No matter where this key pathway functions, these data provide a parallel with human patient data indicating that hypocalcemia is associated with disruptions in auditory gating [27,28,60].

Interestingly, this pathway retains function throughout larval development. We found that *cdh16* regulates acoustic thresholds and habituation after the development of the acoustic startle circuit and after the CS is established. Restoration of *cdh16* expression at 5 and 6 dpf reverts behavioral deficits such that responding is normal later on day 6. Similarly, *pappaa* function is sufficient later in development. Restoration of PI3K signaling downstream of *pappaa* at 5 dpf restores habituation [11]. Low-Ca^2+^ exposure also causes hypersensitivity independent of early development: acoustic hypersensitivity is apparent after only 4 h in low Ca^2+^ media in 5 dpf fish. Similarly, in patients with hypocalcemia, psychotic symptoms are locked to periods of Ca^2+^ dysregulation, and normalization of Ca^2+^ levels can normalize symptoms [27,28]. These data extend previous findings that developmental exposure to Cadmium (an inhibitor of Ca^2+^ channel function) impacts sensory thresholds [61], indicating that even acute disruptions in Ca^2+^ homeostasis can impact behavior.

We do not yet know precisely how hypocalcemia impacts activity within the neuronal circuits responsible for gating sensory stimuli [62]. In hippocampal slices, low Ca^2+^ exposure results in an increase in spontaneous neuronal activity [63]. This effect may be partially explained by a somewhat depolarized resting membrane potential mediated by depolarizing currents through sodium leak channels (NALCN) under conditions of hypocalcemia [64]. In this model, Ca^2+^ is detected by the calcium sensing receptor CaSR, which suppresses current through NALCN [64]. Under conditions of low Ca^2+^, NALCN currents are dis-inhibited and neurons are somewhat depolarized. Signaling through CaSR separately regulates firing frequency through regulation of calcium-activated potassium channels [65].

Interestingly, loss-of-function mutations in the Calcium sensing receptor, *casr*, were also uncovered in the forward genetic screen for regulators of acoustic startle response gating [12]. Like Cdh16, CaSR regulates whole-body Ca^2+^ levels, but in humans, patients with inactivating mutations in *casr* are hypercalcemic [66] (in contrast to *cdh16* mutants, which we showed are hypocalcemic). Mirroring their opposing impacts on Ca^2+^ homeostasis, CaSR and Cdh16 have somewhat opposing impacts on behavior. While *cdh16* mutants are hypersensitive to acoustic stimuli and perform more short-latency startles, *casr* mutants perform fewer short-latency startles, instead responding to acoustic stimuli by primarily performing a distinct behavior, the long-latency C-bend, which wild type zebrafish larvae ordinarily perform in response to lower-intensity stimuli [12]. However, the role of CaSR is likely more complex. In addition to regulating serum Ca^2+^, CaSR functions in neurons to regulate acoustic startle response gating [16]. Restoration of CaSR function in *casr* mutant animals in a small population of hindbrain neurons that project in the vicinity of the Mauthner cell restores normal startle responsiveness [16]. Presumably, these rescued animals remain hypercalcemic, but rescue of CaSR signaling within this particular population is sufficient to normalize behavior. How and if the Cdh16, Stc1l, Papp-aa pathway interacts with CaSR signaling in the brain is not yet known, though we note that Papp-aa is expressed in multiple neuronal populations within the acoustic startle circuit [11,25] and could interact with CaSR there.

Additional support for a link between the *pappaa* and *casr* pathways is provided by our recent work finding similar whole-brain activity patterns and drug response profiles for animals carrying loss-of-function mutations in *pappaa* and *ap2s1* [17], which genetically interacts with *casr* [12]. Like *pappaa* and *cdh16*, *ap2s1* mutants are hypersensitive and fail to habituate to acoustic stimuli [11,67], and mutations in *ap2s1* significantly suppress the CaSR phenotype [12]. In light of our new data connecting *pappaa* to *cdh16* and Ca^2+^ homeostasis, and *ap2s1*’s genetic interaction with the Ca^2+^-regulatory CaSR, we now propose that the commonalities between the *ap2s1* and *pappaa* whole-brain activity patterns may reflect common dysregulation of Ca^2+^.

*cdh16* and *pappaa* mutants, as well as wild type animals exposed to low Ca^2+^, show acoustic sensory gating deficits. Conversely, only low Ca^2+^-exposed fish and *pappaa* mutants exhibit deficits in the visually evoked O-bend response. Interestingly, in addition to its expression in ionocytes and neuromast supporting cells, *pappaa* is expressed in the retina, where mutants show disrupted development of synapses between photoreceptor cones and OFF bipolar cells [49]. *pappaa* mutants also have a thinner outer plexiform layer (the layer where cones make synaptic contacts with bipolar cells) [49]. Notably, in mice and zebrafish, mutations in *cacna1fa,* which encodes a Ca^2+^ channel essential for maintaining resting Ca^2+^ currents in photoreceptors, are also associated with visual defects and thinning of the outer plexiform later. Mutations in *pde6c*, which regulates Ca^2+^ channels in cones, are similarly associated with both visual defects and defects in the outer plexiform layer [68–70]. Finally, acute exposures of dissected mouse retinae to Ca^2+^ chelators results in disassembly of presynaptic terminals in photoreceptors [71], and acute inhibition of Ca^2+^ channels results in synaptic deficits in the zebrafish retina [68]. These data, together with our finding that low Ca^2+^ and loss of *pappaa* have the same effects on the response to dark flash, lead us to propose that disruptions in Ca^2+^ homeostasis may be responsible for the *pappaa* visual and acoustic phenotypes.

### Limitations

Although we demonstrate a genetic relationship between *cdh16, stc1l*, and *pappaa*, we do not determine a mechanism through which Cdh16 and Stc1l affect Papp-aa. Prior work has shown that loss of *stc1l* results in increased *pappaa* expression [23]. Although we would have predicted that *cdh16* mutants, which overexpress *stc1l,* would therefore have decreased *pappaa* expression, we find that *pappaa* expression at the whole-animal level is not affected via RT-qPCR. This leaves open the possibility that Cdh16 and Stc1l regulate Papp-aa by regulating its enzymatic activity. Indeed, *in vitro* experiments demonstrate that Stc suppresses Papp-aa enzymatic activity [47,48]. A second possibility is that Cdh16 and Stc1l influence *pappaa* expression in a regionally-restricted manner not detected by our RT-qPCR experiments. Future work could begin to disentangle these mechanisms by assessing *pappaa* expression using *in situ* hybridization to visualize region-specific changes in *cdh16* mutants, for example in the retina versus in the ionocytes.

Although we find no evidence that *cdh16* is expressed in the brain, we did not test cell-type specific rescue for *cdh16* within the CS in this study. It therefore remains possible that *cdh16* could function outside of the CS. Nonetheless, our findings that *cdh16* is expressed in the CS throughout embryonic and larval development, that it suppresses *stc1l* expression, including by regulating CS size, that overexpression of *stc1l* alone recapitulates the *cdh16* mutant phenotype, and that CS ablations cause hyposensitivity, strongly support a model in which *cdh16* functions in the CS to regulate acoustic startle thresholds in larval zebrafish.

We observe some complexity in the relationship between environmental Ca^2+^ and sensitivity to acoustic stimuli and in the relationship between environmental Ca^2+^ and whole-body Ca^2+^. First, while our lowest Ca^2+^ concentration causes hypersensitivity, 0.02 mM surprisingly causes hyposensitivity. We speculate that this unexpected result may reflect engagement of compensatory mechanisms that drive animals toward hyposensitivity. However, why such compensatory mechanisms are not engaged in 0.001 mM Ca^2+^ is not clear. We note that wild type zebrafish larvae show a remarkable ability to cope with low environmental Ca^2+^ in terms of maintaining bone-mineralization [50]. However, the specific mechanism underlying the complexity in the behavioral response to lowered Ca^2+^ remains unexplained. Secondly, while exposure to high environmental Ca^2+^ caused a significant increase in whole-body Ca^2+^ as measured by our colorimetric assay, low Ca^2+^ trended toward but did not cause a significant decrease. We speculate that the subtle effect of reducing environmental Ca^2+^ may reflect a limitation of our assay: perhaps it largely measures Ca^2+^ reserves in bone, which is present by 5 dpf, and which might outweigh fluctuations in serum Ca^2+^.

Taken together, our studies support a model in which Cdh16 suppresses Stc1l both by suppressing the expression of *stc1l* and proliferation or survival of Stc1l-expressing cells in the CS. Cdh16 continues to play this role throughout larval development rather than during the specification or assembly of the CS. In *cdh16* mutants, overexpressed Stc1l functions to suppress Papp-aa and ionocyte proliferation, and ultimately promotes hypocalcemia and hyper-responsiveness to acoustic stimuli. This work highlights a previously unappreciated role for Ca^2+^ homeostasis in the regulation of acoustic response thresholding and identifies a new brain non-autonomous pathway for the regulation of behavior.

## Materials and methods

### Ethics statement

All procedures were approved by the University of Colorado Anschutz Medical Campus School of Medicine Institutional Animal Care and Use Committee (IACUC). Protocol #1,127. The CU Anschutz IACUC follows the US Public Health Service’s Policy on Humane Care and Use of Laboratory Animals and Guide for the Care and Use of Laboratory Animals.

### Experimental model and subject details

Zebrafish larvae were obtained from pairwise or group crosses of adult zebrafish carrying mutations or transgenes of interest on the TLF (WT) background. Larvae were raised at 28.5 °C in E3 media and sorted for normal development.

The *p173* allele of *cdh16* and the *p170* allele of *pappaa* were recovered from a forward genetic screen [11]. Mutants were genotyped using proprietary allele specific primer sequences (LGC genomics) and the KASP assay method, which utilizes FRET to distinguish between alleles. For genotyping of *p173* in the context of *Tg[hsp70:cdh16-p2a-mkate],* CAPS primers 107 and 108 were used in combination with MseI (see Table 1).

**Table 1:** Genotyping.

Allele	Primer #	Primer sequence	Annealing temp (°C)	Extension time (s)	Restriction enzyme	Expected amplicon size (bp)
*cdh16* ^*co79*^	657	CACTTGGTTTATTGCACTGAGCg	58	30	NcoI-HF	Wild type: 36, 77 and 118; Mutants: 77 and 154
	658	ccttgcagaaggaactcacCTTG				
*cdh16*^*p173*^ *in Tg[hsp70:cdh16-p2a-mkate]*^*co113*^ background	107	GTAACTCCTCTCTGTCCGCC	54	30	MseI	Wild type: 278; Mutants: 195 and 83
	108	gctattgctcaacaggtggaa				
*cdh16* ^*co120*^	931	GCTTTCTGAGAGTGCTCATCTT	57	30	N/A	Wild type: 95; Heterozygotes: 95, 129; Mutants: 129
	932	CTGCCGTGATGGTGAGTGTG				
	658	ccttgcagaaggaactcacCTTG				
*pappaa* ^*p170*^	562	Proprietary allele-specific primers, LGC Genomics	N/A			
*cdh16* ^*p173*^	665	Proprietary allele-specific primers, LGC Genomics	N/A			

*co79* and *co120* mutant alleles were generated using CRISPR-Cas9 mutagenesis. To create these alleles, wild type embryos were injected with either sgRNA 622 (*co79*) or sgRNAs 622 and 867 (*co120*) (Tables 1 and 2). sgRNAs were designed using ChopChop [72], purchased from IDT, and reconstituted to 200 μM using the IDT-provided duplex buffer. sgRNAs were combined with tracrRNA, also purchased from IDT, to form a 50 μM duplex by heating at 95 °C in a thermocycler for 5 min, followed by cooling to RT for 10 min. Injection mixes were prepared by mixing 1 μL of 50 μM duplex together with 1 μL Cas9 protein (5 mg/mL) obtained from PNA Bio and 1 μL phenol red. *cdh16*^*co79*^ mutations were genotyped by PCR with primers 657 and 658 (Table 1). *cdh16*^*co120*^ mutations were genotyped via PCR with primers 931, 932, and 658 (Table 1).

**Table 2:** Analyzing CRISPR efficiency.

Crispant	Guide #	Target sequence	Primer #	Primer sequence	Annealing temp (°C)	Extension time (s)	Expected amplicon size (bp)
*stc1l_5'*	942	CCGTGCTCGTCTCGGTTGTG	951	GTAAAGTTGCAGACATGCTCCTG	59	30	228
			952	gccacagtgcttaccactgag			
*stc1l_middle*	943	AGCATCAAGTGCATGGCCAA	953	GGAGTGGAAGTTTGGCCAATG	59	30	129
			954	GGTCTGGAACACTTTGGAGGTG			
*stc1l_3′*	944	CTCAGTCCCAGAAGGCTCGG	955	CCACACTCTTCCAGCTGCTTC	59	30	155
			956	GGCGAACAGGTGAGTCTGG			
*stc1l_5′ > middle*	942	CCGTGCTCGTCTCGGTTGTG	951	GTAAAGTTGCAGACATGCTCCTG	59	30	123 (mutant only)
	943	AGCATCAAGTGCATGGCCAA	954	GGTCTGGAACACTTTGGAGGTG			
*stc1l_middle > 3′*	943	AGCATCAAGTGCATGGCCAA	953	GGAGTGGAAGTTTGGCCAATG	59	30	194 (mutant only)
	944	CTCAGTCCCAGAAGGCTCGG	956	GGCGAACAGGTGAGTCTGG			
*stc1l_5′ > 3′*	942	CCGTGCTCGTCTCGGTTGTG	951	GTAAAGTTGCAGACATGCTCCTG	59	30	188 (mutant only)
	944	CTCAGTCCCAGAAGGCTCGG	956	GGCGAACAGGTGAGTCTGG			
*cdh16_5′*	622	ACTATGATGGTATTTTCCCA	657	CACTTGGTTTATTGCACTGAGCg	58	30	231
			658	ccttgcagaaggaactcacCTTG			
*cdh16_middle*	623	CTGGCTGAGGACTCGTCGGT	869	GCTGCCGATAATGACGATCCG	59	30	147
			870	AGTTCCCTCCATGCTGTCTG			
*cdh16_3′*	867	TTTCGTGTGGACCGGGACTC	659	gtttctgcagTACGGCCCATTC	59	30	129
			646	ATTCAAGCCTGTAGTCCACCTG			
*cdh16_5′ > middle*	622	ACTATGATGGTATTTTCCCA	657	CACTTGGTTTATTGCACTGAGCg	59	30	237 (mutant only)
	623	CTGGCTGAGGACTCGTCGGT	870	AGTTCCCTCCATGCTGTCTG			
*cdh16_middle > 3′*	623	CTGGCTGAGGACTCGTCGGT	869	GCTGCCGATAATGACGATCCG	59	30	191 (mutant only)
	867	TTTCGTGTGGACCGGGACTC	646	ATTCAAGCCTGTAGTCCACCTG			
*cdh16_5′ > 3′*	622	ACTATGATGGTATTTTCCCA	657	CACTTGGTTTATTGCACTGAGCg	59	30	281 (mutant only)
	867	TTTCGTGTGGACCGGGACTC	646	ATTCAAGCCTGTAGTCCACCTG			

Transgenic animals carrying *Tg[hsp70:cdh16-p2a-mkate]* (*co113*) were generated by cloning the *cdh16* cDNA from total zebrafish RNA at 5 dpf into *pME-cdh16-p2a-mKate*. Gateway cloning was used to recombine *pME-cdh16-p2a-mKate* into a pDest vector containing the *hsp70* promoter and I-SceI restriction sites, generating *hsp70-cdh16-p2a-mKate*. I-SceI transgenesis was performed as previously described [73] by injecting I-SceI and the *hsp70-cdh16-p2a-mKate* plasmid into 1-cell stage TLF embryos. *G*_0_ injected larvae were raised, outcrossed, and heat-shocked at 37 °C in a thermocycler for 45 min to identify carriers. Larvae expressing the transgene were identified by screening for mKate using a fluorescent stereomicroscope (Leica M205FCA). For behavior experiments, animals were pre-screened for fluorescence and genotyped post-hoc using primers 107 and 108 (Table 1).

Transgenic animals carrying *Tg[hsp70-V5-stc1l]* were generated by cloning V5-*stc1l* (obtained as a custom gene block from IDT) (Table 3) to generate *pME-V5-stc1l* through HiFi DNA Assembly. Gateway cloning was then used, as described above, to recombine *pME-V5-stc1l* into the pDest vector containing the *hsp70* promoter and I-SceI restriction sites, to generate *hsp70*-*V5*-*stc1l*. I-SceI transgenesis was performed as described above; we injected either *hsp70* vector alone or *hsp70-V5-stc1l*. *G*_0_ embryos were raised to 4 dpf and heat-shocked at 37 °C in a thermocycler for 45 min. Behavior was then tested at 5 dpf. Presence of *hsp70-V5-stc1l* was confirmed after behavior testing using cloning primer 1,033 (5′-CTTGTTCTTTTTGCAGgccaccATGCTCCTGAAAAGCGGATTTC-3′) and genotyping primer 706 (5′-CAGCAGAGGGTTTGGGATAG-3′), (annealing temperature: 56 °C, extension time: 30 s, product size: 118 bp). All *G*_0_ larvae injected with the *hsp70-v5-stc1l* construct genotyped positive; all that were injected with the control plasmid genotyped negative.

**Table 3:** Cloning primers.

Primer/gBlock Name	Primer/gBlock #	Primer/gBlock sequence
773_cdh16_IF_vector	773	CTTGTTCTTTTTGCAGGATgccaccATGGAATATGTGAGCACTTGGT
776_cdh16_IF_p2A	776	ACTGAAGTTCGTGGCCAGAGACACATTGAGCGGCACC
981_stc1L_v5_gBlock	981	CTTGTTCTTTTTGCAGATGCTCCTGAAAAGCGGATTTCTTTTGCTGGTGGTTTTGGCTGCTTGTGCATTTTGCACAACTCAGGAATCCACAGGCAAGCCTATCCCAAACCCTCTGCTGGGCCTGGACTCCACAAGACAACCGAGACGAGCACGGTTCTCATCCAACACCCCTTCTGATGTTGCCCGCTGTCTGAATGGCGCTCTGCAAGTGGGTTGTGCGACCTTCGCATGTCTGGAAAACTCCACCTGCGACACCGACGGCATGCACGAGATCTGCAACGTCTTCCTCCACACAGCTGCTGTTTTTAACACAGAGGGTAAAACATTTGTGAAAGAGAGCATCAAGTGCATGGCCAACGGCATCACCTCCAAAGTGTTCCAGACCATTAAGCGCTGCTCCACCTTCCAGAAGATGATCGCTGAAGTGCAGGAGGAGTGTTATAAGAAGCTGGACCTCTGTGAAGTGGCCCGATCAAACCCTGAGGCCATTGGAGACGTGGTGCAGGTGCCCAACACTTTCCCCAACAGGTATTACAGCACACTTCTGCAGAGTTTAATGGAGTGCGAGGAGGACACAGTGGAGGTGGTTCGAGCTGGTCTGGTGTCCAGACTGGGACCGGATATGGCCACACTCTTCCAGCTGCTTCAAAACAAACCCTGCTCATCCGAACCCGCAGCCGCCGAGCCTTCTGGGACTGAGAGTCAAACCGGCTTCCGCTGGCCCCCAATGTTCAAGATCCAGCCAAACATGTACAACAGAGACCAGACTCACCTGTTCGCCAGAAAACGCTCCATCGTGGGAAGTCCTTAACAAGGCCTCTCGAGC

Transgenic animals carrying the *gal4* driver *Tg[gffDMC130a]* were provided by the lab of Dr. Koichi Kawakami [74]. Transgenic animals carrying *Tg[UAS:Gap43-citrine]* were provided by the lab of Dr. Jonathan Raper [75]. Animals carrying *Tg[her6:mCherry]* [55] were provided by the lab of Dr. James Nichols and outcrossed to TLF for ablation experiments.

To generate conceptual translations of each allele, SMART domain-prediction software was used [76]. SMART identified Cadherin repeats 1–6 based on the full-length protein sequence. Cadherin repeat 7 was not originally identified, however SMART identified a seventh cadherin repeat when the final portion of the extracellular domain was searched alone.

### Behavior testing

Before testing their response to acoustic and visual stimuli, larvae were acclimated to the behavior room inside an incubator kept at 28 °C for 30 min. To measure acoustic startle thresholds, six increasingly intense acoustic stimuli were administered 5 times each, 40 s apart, after which acoustic startle response habituation was measured by providing 40 stimuli with a 3-s inter-stimulus interval (ISI). Visual motor responses (VMR) were measured by first dark-acclimating larval zebrafish inside the behavior arena. Next, the lights were turned on for a 7-min period to assess initial visual motor reactivity in response to light. Then, the lights were turned off for 7 min to assess the initial visual motor response to darkness. Light flash reactivity was examined by first dark-acclimating larval zebrafish inside the behavior arena. Next, larvae were exposed to 10 pulses of light with a one second duration, 30 s apart. To assess dark flash reactivity, 6 dpf larvae were acclimated to the light inside the behavior arena. Following this, the lights were extinguished 5 times in pulses lasting 1 s with a 1-min ISI. To assess dark-flash habituation 60 additional dark flash stimuli were administered with a 10-s ISI. During these final stimuli, the camera recorded behavior during every other stimulus. For the above-described behavior assays, larvae were loaded onto a custom-made acrylic 6 × 6 well-plate attached to a mini-shaker (Brüler and Kjær, Model 4,810), which was used to deliver the acoustic stimuli. A cover was placed over the rig for assays of visually evoked behaviors.

Behavior was recorded with a high-speed camera (FASTCAM Mini UX50 Type 160K-M-32G) placed above the plate and an LED light pointed at the behavior arena was used for light stimuli. Acoustic stimuli were calibrated using an accelerometer (PCB Piezotronics, Y355B03) and stimulus intensities are reported in *g* or acceleration due to gravity. To analyze behavior, video files were background-subtracted and then analyzed using FLOTE, Batchan [29], and Microsoft Excel. Statistical analyses and graphing were performed using Graphpad Prism.

Larvae were tested for thermal behavior using a 96-well (square wells) plate loaded into a DanioVision observation chamber running EthoVision XT 11.5 software (observation chamber and software, Noldus, Leesburg, VA). The temperature in the observation chamber was set using a temperature control unit. Larvae were acclimated to the baseline temperature of 28.5 °C for 30 min, after which their total distance moved was recorded for 2 min. The temperature was then raised to 33.5 °C, and fish were recorded again for 2 min. All behavioral assays were performed at 5 dpf, except for our dark flash assay, which was performed at 6 dpf.

### Heat-shock induced *cdh16* rescue and *stc1l* overexpression

To induce expression of *hsp70-cdh16-p2a-mKate*, zebrafish embryos or larvae were placed in a 96-well plate at a density of no more than 5 larvae per well. The plate was heated to 37 °C for 45 min using a thermocycler. Larvae were then recovered to petri dishes for at least 5 h before behavior testing. Similarly, to overexpress *stc1l* via *hsp70-V5-stc1l*, 4 dpf larvae were placed into a 96-well plate at a density of no more than 5 larvae per well. The plate was heated to 37 °C for 45 min using a thermocycler. The larvae were then recovered to petri dishes for 24 h before testing at 5 dpf.

### Crispant (*F*_0_) mutagenesis and behavior analysis

sgRNAs targeting *cdh16* (622, 623, 867) and *stc1l* (942, 943, 944) were designed using ChopChop [72]. Scrambled sgRNAs (759, 760, and 761) were used as controls and were designed by IDT as previously described [51]. The sgRNAs were purchased from IDT and reconstituted to 200 μM stocks using the IDT-provided duplex buffer. sgRNAs were then combined individually with tracrRNA, also purchased from IDT, to form a 61 μM duplex by heating at 95 °C in a thermocycler for 5 min, followed by cooling to RT for 10 min. Injection mixes were prepared by mixing 1 μL of duplex together with 1 μL Cas9 nuclease V3 (10 μg/μL; IDT Cat #1,081,059). 1nl of injection mix was injected in the yolk at the single cell stage, before the cell inflates.

The mutation rate in crispants was assessed by PCR using primers flanking the sgRNA target sequences to detect indels and large deletions. Following behavioral analysis, we genotyped larvae injected with gene-specific sgRNAs and larvae injected with control sgRNAs to confirm guide efficiency (see Tables 2 and 4).

**Table 4:** CRISPR guide cutting efficiency.

Mutagenesis	Fig 6A (*stc1l* to WT) rates	Fig 6C (*stc1l* to *cdh16*) rates	Fig 6D (*stc1l* to *pappaa*) rates	Fig 6E (*cdh16* to *pappaa*) rates
**Overall rate**	**90.91%**	**100%**	**100%**	**100%**
Insertion/deletion (Indel) rate	90.91%	100%	100%	100%
Large deletion rate	81.82%	81.82%	100%	100%
5′ guide rate	90.91%	100%	100%	90.91%
Mid guide rate	90.91%	90.91%	100%	100%
3′ guide rate	0%	0%	0%	100%
5′-mid rate	63.64%	81.82%	81.82%	63.64%
Mid-3′ rate	27.27%	9.09%	54.55%	63.64%
5′-3′ rate	18.18%	45.45%	54.55%	54.55%

### Hybridization chain reaction (HCR) FISH staining

HCR probes, hairpins, and buffers were purchased from Molecular Instruments. Staining for experiments utilizing *cdh16, cdh16 (deletion set), stc1l,* and *trpv6* HCR probes was performed using the manufacturer’s protocol: “HCR RNA-FISH protocol for whole-mount zebrafish embryos and larvae (*Danio rerio*)” with the following modifications: we did not apply PTU to inhibit melanogenesis, except in the case of *trpv6* HCR to quantify NaR cells, we used 30 larvae per Eppendorf tube, and we used 8 μl of 1 μM *cdh16* and *cdh16 (deletion set)* probe solutions, all other probes were used at 4 μl of 1 μM probe set solution in 500 μL of Probe Hybridization Buffer. For CS imaging, animals were mounted laterally in 1.5% low-melt agarose in PBS and imaged using a 63× objective on a 3i Marianas Spinning Disk Confocal Microscope. For ionocyte imaging, animals were mounted with their ventral side facing the objective. For whole-fish images, 8 individual 20× images were acquired to capture the entire larva using the 8 × 1 montage function in SlideBook and then stitched using the legacy montage function.

### Calcium manipulations

To create Ca^2+^-supplemented media, we first created a stock solution of 60× E3 embryo media without Ca^2+^: 300 mM NaCl, 10.2 mM KCl, and 19.8 mM MgSO_4_·7H_2_O. A separate stock solution of 60× CaCl_2_·2H_2_O (Sigma CAS#:10035-04-8) was also made. Ca^2+^ concentrations of 10 mM Ca^2+^, 0.33 mM (Normal), 0.02 mM, and 0.001 mM were generated by mixing 60× E3 and 60× CaCl_2_ in the appropriate ratios. At 5 dpf, larvae were rinsed three times out of E3 media containing normal Ca^2+^ (0.33 mM Ca^2+^), and into one of the four different Ca^2+^-supplemented media concentrations four hours before performing behavior and then tested in those same Ca^2+^ concentrations.

### Corpuscle ablations

*Tg*[*her6:mCherry]* embryos were screened for mCherry expression at 3 dpf using a fluorescent stereomicroscope. Four dpf mCherry-positive larvae were live-mounted laterally in 1.5% low-melt agarose (Lonza Cat# 50,101) in E3 embryo media on a 3.5 cm glass-bottom dish. The CS were identified and then ablated using 532 nm pulse laser attached to a 3i Marianas spinning disk confocal microscope with a 63× objective. To ensure complete ablation, an average of 3 laser pulses were administered per corpuscle (laser pulses were delivered until the CS was eliminated). For sham ablations, a target region posterior to the kidney and yolk extension was located and ablated, after which the CS were re-located and confirmed to be undamaged. Ablated and sham-ablated larvae were then unmounted and placed in a 6 cm petri dish with fresh E3 to recover for approximately 21 h, after which they were behavior tested at 5 dpf for acoustic startle thresholds and habituation.

### WGS and molecular cloning of *cdh16*

Molecular cloning of the *cdh16* allele was performed as previously described [11,14]. Pools of 50 behaviorally identified *p173* mutant larvae were collected and used to prepare genomic DNA (gDNA) libraries. gDNA was sequenced with 100-bp paired-end reads on the Illumina HiSeq 2000 platform, and homozygosity analysis was done using 463,379 SNP markers identified by sequencing gDNA from ENU-mutagenized TLF and WIK males as described previously [11].

### Calcium content assays

Whole-body Ca^2+^ was quantified using a colorimetric assay kit (Abcam ab102505). For the first experiment measuring Ca^2+^ in *cdh16*^*p173*^, 2 dpf larvae were live tail-clipped and genotyped for the *cdh16*^*p173*^ allele. At 4 dpf, 10–15 larvae were pooled in each of 6 Eppendorf tubes: three WT biological replicates and three mutant biological replicates. For the second experiment, Ca^2+^ was measured following acute (four hour) exposure of wild type larvae to four Ca^2+^ concentrations at 5 dpf (10 mM, 0.33 mM, 0.02 mM, 0.001 mM). Then 10–15 larvae were pooled in 12 Eppendorf tubes: three WT biological replicates for each of the four Ca^2+^ concentrations. The assays were then performed as previously described [50].

### RT-qPCR

Larvae were dissected to remove the distal tip of the tail for genotyping. To generate cDNA from heads and trunks, larvae were dissected to isolate the head from the trunk at the base of the hindbrain. Tissue to be used for RT-qPCR was placed into RNAlater (Sigma Cat# R0901-100ML) and stored at 4 °C. Following genotyping, whole larvae (Figs 5A and 6F), trunks (Fig 5B), or heads (Fig 5C) were pooled by genotype and total RNA was extracted using Trizol/Chloroform followed by the RNeasy Plus Mini Kit (Qiagen Cat# 74,143). cDNA pools were generated using SuperScript II Reverse Transcriptase (Invitrogen Cat# 11,904−018). qPCR was performed with LUNA qPCR MasterMix (NEB Cat# M3003) on a QuantStudio 3 Real-Time PCR System (Fisher Cat# A28566) using qPCR primers (Table 5) designed for each target gene. Expression levels of target genes were normalized to *gapdh*.

**Table 5:** qPCR primers.

Target gene	Primer #	Sequence
*gapdh*	809	TGCTGGTATTGCTCTCAACG
*gapdh*	810	AACAGCAAAGGGGTCACATC
*pappaa*	805	AGACCAGCTGAGACTCAAGCC
*pappaa*	806	CATCCACGATCACTAGAGGCG
*stc1l*	985	CCAGCTGCTTCAAAACAAACC [23]
*stc1l*	986	ATGGAGCGTTTTCTGGCGA [23]

### Analysis of *stc1l* in the corpuscles of Stannius

To measure *stc1l* fluorescence intensity, images acquired at 63× magnification of the *stc1l*-labeled CS were converted to OME-TIFFs. TIFFs were sum projected over their entire *z*-stack using FIJI. Regions of interest were then drawn freehand around the borders of each corpuscle, or around the border of the entire structure when the two corpuscles overlapped in Z. Fluorescence intensity was then measured and divided by the number of corpuscles (2 CS were observed in every animal except one mutant that had 3 CS). To count the total number of *stc1l+* cells in the CS, *z*-stacks of the CS were visually inspected using Slidebook imaging software and marking each cell using the freehand ROI tool to assign it a number. This number was divided by the number of corpuscles, to quantify the number of *stc1l+* cells per CS. Finally, the intensity per CS was divided by the number of *stc1l+* cells per CS, to quantify the relative intensity of *stc1l* signal per CS cell.

### Epithelial ionocyte (NaR cell) quantification

Two *z*-stacks encompassing *trpv6* expression in NAR cells along the jaw and the skin overlying the swim bladder were acquired at 20× magnification for each larva. To quantify the number of NaR cells, we stitched swim bladder and jaw images using FIJI. A maximum projection was then made from the combined *z*-stack and the despeckle function was used to reduce noise. An image threshold was applied with 6,725 as the minimum value and 65,535 as the maximum value. The watershed function was then used to further delineate the cell borders from one another. When necessary, an ROI was drawn to exclude any autofluorescence in the eyes, and the included region was analyzed for the total number of particles, taken to be the total number of NaR cells in this region.

### Mauthner morphology quantification

To assess Mauthner morphology, WT and *cdh16*^*p173*^ mutant larvae heterozygous for each of *Tg[Gap43:Citrine*] and *Tg[Gffdmc130a]* were fixed overnight in sweet fix (4% paraformaldehyde, 4% sucrose, 1× PBS). After 3 washes in PBT, tails were clipped, lysed, and KASP genotyped for the *cdh16*^*p173*^ allele. Brains were then dissected from wild type and *cdh16* mutant larvae, mounted dorsal side down in Vectashield, and assigned random numbers by an independent investigator to ensure blinding during imaging and analysis. *Z*-stacks of Mauthner neurons were acquired using a 3i Marianas spinning disk confocal microscope with a 63× objective. Maximum intensity projections of the soma and lateral dendrites were generated in FIJI and measurements were performed using the straight-line tool. Mauthner neuron morphology was quantified as follows: Whole Mauthner length was measured from the axon initial segment to the distal-most tip of the lateral dendrite. Soma length was measured from the axon initial segment to the first narrowing at the soma-lateral dendrite boundary. Lateral dendrite length was measured from this point to the distal-most tip of the distal-most lateral dendrite branch. Two Mauthner neurons were measured and displayed per fish, except in cases where only one Mauthner neuron was labeled (for WT, *n* = 13 showed labeling in both Mauthners, *n* = 3 had only one labeled; for *cdh16* mutant, *n* = 9 showed labeling in both Mauthners, *n* = 1 had only one labeled).

### Quantification and statistical analysis

Statistical tests were performed in Graphpad PRISM 9 and 10. To determine normality for each data set, the D’Agostino and Pearson test was performed. In normally distributed data, an unpaired *t* test, one-way ANOVA, or two-way ANOVA was performed as needed. To account for multiple comparisons in two-way ANOVAs, the Šidák’s multiple comparisons test was performed when comparing means across one variable while the Tukey’s multiple comparisons test was used to compare means between all experimental groups. In datasets that are not normally distributed, a Mann–Whitney test was executed to compare two groups and a Kruskal–Wallis test was used to compare between greater than two groups.

### Whole brain morphometric imaging and analysis

Six dpf larvae (*n* = 60) from a *cdh16*^*p173*^ heterozygote incross were acclimated to the behavior testing room for 30 min. Following acclimation, the larvae were placed in a cell strainer within a 6 cm petri dish containing E3 for 30 min. Finally, spontaneous behavior was recorded for 16 min before the cell strainer was removed and placed into a 6 well dish containing 4% paraformaldehyde in PBT (PBS-Triton 0.25%) for 45 s to flash-fix the larvae. The cell strainer was then transferred to a solution of 4% paraformaldehyde in PBS, incubating at 4 °C overnight. Larvae were moved from the cell strainer to a 1.5 mL tube and washed with PBT for three, 5-min washes. To increase the ratio of mutants to WT larvae included in the imaging experiment, tail clips were collected from each sample, lysed, and KASP genotyped for the *cdh16*^*p173*^ mutation. Wild type and mutant larvae were pooled at a 1:1 ratio into a 1.5 mL tube containing PBT and stained according to a previously developed immunohistochemistry protocol for MAP-mapping [40] with procedural alterations [17]. Finally, samples were mounted onto a glass-bottom dish using 1.5% low-melt agarose made with PBS. Each larva was positioned with the dorsal portion of its brain facing the glass bottom of the dish. Whole-brain *z*-stacks were collected for each sample using an LSM780 microscope with a 20× objective and 2 × 1 tile scanning. Larvae were unmounted from the agarose and gDNA was prepared for KASP genotyping. Morphometric analysis of *cdh16*^*p173*^ mutants was then performed as previously described [39,40]. Differences in whole brain morphology were examined by assessing the significant delta medians of mutants over WT.

## Supporting information

S1 Fig Whole-brain morphometric analysis reveals minimal changes to region-by-region brain volume. **(A)** Summary of whole-brain morphometric data for 6 dpf *cdh16*^*p173*^ mutants (*n* = 13) as compared to siblings (*n* = 16). Region-by-region differences in volume are indicated in yellow (regions that are larger in mutants) or cyan (regions that are smaller in mutants). Image is a summed stack of the significant delta medians of mutants over wild types. Note there are no colored pixels within the brain, indicating no significant differences between mutants and siblings across the annotated brain regions.
(TIF)

S2 Fig Cadherin-16 is expressed in the CS. **(A)** Schematic of the large (approximately 49 kB) deletion allele that we generated in the *cdh16* locus (*cdh16*^*co120*^). **(B)** *in situ* HCR image of *cdh16*^*co120*^ mutant larva using only probes that bind within the large deletion. The corpuscles of Stannius are not labeled. All other expression, including weak labeling in the head, can be considered background (representative image in Fig 4A). Shown is a representative image of *cdh16*^co120^; *n* = 4 larvae imaged.
(TIF)

S3 Fig Cadherin-16 does not regulate Mauthner cell morphology. **(A–E)** Analysis of the Mauthner neurons responsible for the acoustic startle (SLC) response. **(A–C)** *cdh16*^*p173*^ mutants (*n* = 19) and wild type siblings (*n* = 29) have no differences between Mauthner soma length **(A)** *p* = 0.3169, lateral dendrite length **(B)** *p* = 0.4054, or total length **(C)** *p* = 0.9248, unpaired *t* test. Error bars represent SD. **(D–E)** No morphological differences are detected between the Mauthner cells of *cdh16*^*p173*^ mutant (bottom image) and WT larvae (top image). The data underlying this figure can be found in S1 Data.
(TIF)

S4 Fig Ca^2+^ homeostasis and the corpuscles of Stannius regulate sensory gating. **(A–D)** E3 media with differing Ca^2+^ concentrations were applied to WT larvae at 5 dpf. Whole-body Ca^2+^ was measured 4 h later **(A)** or behavioral assays were performed 4 h later **(B–D)**. **(A)** Exposure to high (10 mM) Ca^2+^ caused a slight but significant increase in whole-body Ca^2+^ levels, compared to normal 0.33 mM Ca^2+^ (*p = 0.024). Exposure to media containing low Ca^2+^ (0.02 and 0.001 mM) did not significantly affect whole-body Ca^2+^ levels (*n* = 3 biological replicates per condition, *p* = 0.8297, *p* = 0.093; one-way ANOVA with Dunnett’s multiple comparisons test). Error bars represent SD. **(B)** As is observed in *pappaa* mutant larvae, animals in 0.001 mM Ca^2+^ (*n* = 17) show decreased responding to dark flashes relative to siblings in 0.33 mM Ca^2+^ (*n* = 18) ****p < 0.0001, Kruskal–Wallis test with Dunn’s multiple comparisons test. Error bars represent SD. **(C)** Animals in the lowest (0.001 mM) Ca^2+^ concentration (*n* = 18) were more responsive to the lights-on stimulus in the visual motor assay as compared to their siblings in a normal 0.33 mM Ca^2+^ concentration (*n* = 18), *p* = 0.0033, Kruskal–Wallis test with Dunn’s test for multiple comparisons. Error bars represent SD. **(D)** Animals in 0.001 mM Ca^2+^ (*n* = 18) displayed more robust responses to a light flash than their siblings in 0.33 mM Ca^2+^ (*n* = 18) ***p = 0.0009, Kruskal–Wallis test with Dunn’s test for multiple comparisons. Error bars represent SD. (**E–F)** Images of 5 dpf WT larvae 24 h after either CS ablation (left) or sham ablation (right). Larvae with ablated corpuscles do not have visible pericardial edema. **(G–J)** *stc1l* HCR to visualize the CS after sham ablation **(G, I)** or CS ablation **(H, J)**. Only a few *stc1l*-positive cells are present in the CS region 4 h after CS ablation **(H)**, and *stc1l* expression is strongly reduced. By 24 h post-CS ablation, the structure has partially regenerated **(J)**. Imaged *n* = 10 CS-ablated 4 h post-ablation, *n* = 5 sham-ablated 4 h post-ablation, *n* = 10 CS-ablated 24 h post-ablation, *n* = 5 sham-ablated 24 h post-ablation. The data underlying this figure can be found in S1 Data.
(TIF)

S1 Data Source data. (XLSX)

## Abbreviations

CS: corpuscles of Stannius
dpf: days post-fertilization
gDNA: genomic DNA
hpf: hours post-fertilization
ISI: inter-stimulus interval
SLC: short-latency C-bend
TM: transmembrane.

## References

[1] Koch M. The neurobiology of startle. Prog Neurobiol. 1999;59(2):107–28. doi: 10.1016/s0301-0082(98)00098-7 . 10463792

[2] Kimmel CB, Patterson J, Kimmel RO. The development and behavioral characteristics of the startle response in the zebra fish. Dev Psychobiol. 1974;7(1):47–60. doi: 10.1002/dev.420070109 . 4812270

[3] Marsden KC, Jain RA, Wolman MA, Echeverry FA, Nelson JC, Hayer KE, et al. A Cyfip2-dependent excitatory interneuron pathway establishes the innate startle threshold. Cell Rep. 2018;23(3):878–87. doi: 10.1016/j.celrep.2018.03.095 . 29669291 PMC6642828

[4] Ortiz EA, Campbell PD, Nelson JC, Granato M. A single base pair substitution in zebrafish distinguishes between innate and acute startle behavior regulation. PLoS One. 2024;19(3):e0300529. doi: 10.1371/journal.pone.0300529 . 38498506 PMC10947677

[5] Marsden KC, Granato M. In vivo Ca^2+^ imaging reveals that decreased dendritic excitability drives startle habituation. Cell Rep. 2015;13: 1733–40. doi: 10.1016/j.celrep.2015.10.060 26655893 PMC4680997

[6] Pantoja C, Hoagland A, Carroll EC, Karalis V, Conner A, Isacoff EY. Neuromodulatory regulation of behavioral individuality in zebrafish. Neuron. 2016;91(3):587–601. doi: 10.1016/j.neuron.2016.06.016 . 27397519 PMC4976045

[7] Wolman MA, Jain RA, Liss L, Granato M. Chemical modulation of memory formation in larval zebrafish. Proc Natl Acad Sci U S A. 2011;108(37):15468–73. doi: 10.1073/pnas.1107156108 . 21876167 PMC3174630

[8] Roberts AC, Reichl J, Song MY, Dearinger AD, Moridzadeh N, Lu ED, et al. Habituation of the C-start response in larval zebrafish exhibits several distinct phases and sensitivity to NMDA receptor blockade. PLoS One. 2011;6(12):e29132. doi: 10.1371/journal.pone.0029132 . 22216183 PMC3247236

[9] McDiarmid TA, Bernardos AC, Rankin CH. Habituation is altered in neuropsychiatric disorders—A comprehensive review with recommendations for experimental design and analysis. Neurosci Biobehav Rev. 2017;80:286–305. doi: 10.1016/j.neubiorev.2017.05.028 . 28579490

[10] Chamberlain PD, Rodgers J, Crowley MJ, White SE, Freeston MH, South M. A potentiated startle study of uncertainty and contextual anxiety in adolescents diagnosed with autism spectrum disorder. Mol Autism. 2013;4(1):31. doi: 10.1186/2040-2392-4-31 . 24007557 PMC3844321

[11] Wolman MA, Jain RA, Marsden KC, Bell H, Skinner J, Hayer KE, et al. A genome-wide screen identifies PAPP-AA-mediated IGFR signaling as a novel regulator of habituation learning. Neuron. 2015;85(6):1200–11. doi: 10.1016/j.neuron.2015.02.025 . 25754827 PMC4368495

[12] Jain RA, Wolman MA, Marsden KC, Nelson JC, Shoenhard H, Echeverry FA, et al. A forward genetic screen in zebrafish identifies the G-protein-coupled receptor CaSR as a modulator of sensorimotor decision making. Curr Biol. 2018;28(9):1357-1369.e5. doi: 10.1016/j.cub.2018.03.025 . 29681477 PMC5940496

[13] Santistevan NJ, Nelson JC, Ortiz EA, Miller AH, Halabi DK, Sippl ZA, et al. cacna2d3, a voltage-gated calcium channel subunit, functions in vertebrate habituation learning and the startle sensitivity threshold. PLoS ONE. 2022;17: e0270903. doi: 10.1371/journal.pone.0270903 PMC9282658 35834485

[14] Nelson JC, Witze E, Ma Z, Ciocco F, Frerotte A, Randlett O, et al. Acute regulation of habituation learning via posttranslational palmitoylation. Curr Biol. 2020;30(14):2729-2738.e4. doi: 10.1016/j.cub.2020.05.016 . 32502414 PMC8446937

[15] Meserve JH, Nelson JC, Marsden KC, Hsu J, Echeverry FA, Jain RA, et al. A forward genetic screen identifies Dolk as a regulator of startle magnitude through the potassium channel subunit Kv1.1. PLoS Genet. 2021;17(6):e1008943. doi: 10.1371/journal.pgen.1008943 . 34061829 PMC8195410

[16] Shoenhard H, Jain RA, Granato M. The calcium-sensing receptor (CaSR) regulates zebrafish sensorimotor decision making via a genetically defined cluster of hindbrain neurons. Cell Rep. 2022;41(10):111790. doi: 10.1016/j.celrep.2022.111790 . 36476852 PMC9813870

[17] Nelson JC, Shoenhard H, Granato M. Integration of cooperative and opposing molecular programs drives learning-associated behavioral plasticity. PLoS Genet. 2023;19(3):e1010650. doi: 10.1371/journal.pgen.1010650 . 36972301 PMC10079226

[18] Wagner GF, Guiraudon CC, Milliken C, Copp DH. Immunological and biological evidence for a stanniocalcin-like hormone in human kidney. Proc Natl Acad Sci U S A. 1995;92(6):1871–5. doi: 10.1073/pnas.92.6.1871 . 7892193 PMC42384

[19] Chang AC, Janosi J, Hulsbeek M, de Jong D, Jeffrey KJ, Noble JR, et al. A novel human cDNA highly homologous to the fish hormone stanniocalcin. Mol Cell Endocrinol. 1995;112(2):241–7. doi: 10.1016/0303-7207(95)03601-3 . 7489828

[20] Olsen HS, Cepeda MA, Zhang QQ, Rosen CA, Vozzolo BL, Wagner GF. Human stanniocalcin: a possible hormonal regulator of mineral metabolism. Proc Natl Acad Sci U S A. 1996;93(5):1792–6. doi: 10.1073/pnas.93.5.1792 . 8700837 PMC39860

[21] Wagner GF, Dimattia GE. The stanniocalcin family of proteins. J Exp Zool A Comp Exp Biol. 2006;305(9):769–80. doi: 10.1002/jez.a.313 . 16902962

[22] Schein V, Cardoso JCR, Pinto PIS, Anjos L, Silva N, Power DM, et al. Four stanniocalcin genes in teleost fish: structure, phylogenetic analysis, tissue distribution and expression during hypercalcemic challenge. Gen Comp Endocrinol. 2012;175(2):344–56. doi: 10.1016/j.ygcen.2011.11.033 . 22154646

[23] Li S, Liu C, Goldstein A, Xin Y, Ke C, Duan C. Calcium state-dependent regulation of epithelial cell quiescence by stanniocalcin 1a. Front Cell Dev Biol. 2021;9:662915. doi: 10.3389/fcell.2021.662915 . 33898465 PMC8063699

[24] Hwang P-P. Ion uptake and acid secretion in zebrafish (*Danio rerio*). J Exp Biol. 2009;212(Pt 11):1745–52. doi: 10.1242/jeb.026054 . 19448083

[25] Liu C, Li S, Noer PR, Kjaer-Sorensen K, Juhl AK, Goldstein A, et al. The metalloproteinase Papp-aa controls epithelial cell quiescence-proliferation transition. Elife. 2020;9:e52322. doi: 10.7554/eLife.52322 . 32293560 PMC7185994

[26] Dauber A, Muñoz-Calvo MT, Barrios V, Domené HM, Kloverpris S, Serra-Juhé C, et al. Mutations in pregnancy-associated plasma protein A2 cause short stature due to low IGF-I availability. EMBO Mol Med. 2016;8(4):363–74. doi: 10.15252/emmm.201506106 . 26902202 PMC4818753

[27] Ang AW, Ko SM, Tan CH. Calcium, magnesium, and psychotic symptoms in a girl with idiopathic hypoparathyroidism. Psychosom Med. 1995;57(3):299–302. doi: 10.1097/00006842-199505000-00013 . 7652132

[28] Mehta S, Mehta S. Hypocalcemia masquerading as schizophreniform disorder. Indian J Psychol Med. 2016;38(5):463–5. doi: 10.4103/0253-7176.191386 . 27833232 PMC5052962

[29] Burgess HA, Granato M. Sensorimotor gating in larval zebrafish. J Neurosci. 2007;27(18):4984–94. doi: 10.1523/JNEUROSCI.0615-07.2007 . 17475807 PMC6672105

[30] Burgess HA, Granato M. Modulation of locomotor activity in larval zebrafish during light adaptation. J Exp Biol. 2007;210(Pt 14):2526–39. doi: 10.1242/jeb.003939 . 17601957

[31] Wolman MA, de Groh ED, McBride SM, Jongens TA, Granato M, Epstein JA. Modulation of cAMP and ras signaling pathways improves distinct behavioral deficits in a zebrafish model of neurofibromatosis type 1. Cell Rep. 2014;8(5):1265–70. doi: 10.1016/j.celrep.2014.07.054 . 25176649 PMC5850931

[32] Randlett O, Haesemeyer M, Forkin G, Shoenhard H, Schier AF, Engert F, et al. Distributed plasticity drives visual habituation learning in larval zebrafish. Curr Biol. 2019;29(8):1337-1345.e4. doi: 10.1016/j.cub.2019.02.039 . 30955936 PMC6545104

[33] Emran F, Rihel J, Dowling JE. A behavioral assay to measure responsiveness of zebrafish to changes in light intensities. J Vis Exp. 2008;(20):923. doi: 10.3791/923 . 19078942 PMC2879884

[34] Gau P, Poon J, Ufret-Vincenty C, Snelson CD, Gordon SE, Raible DW, et al. The zebrafish ortholog of TRPV1 is required for heat-induced locomotion. J Neurosci. 2013;33(12):5249–60. doi: 10.1523/JNEUROSCI.5403-12.2013 . 23516290 PMC3893356

[35] Wendeler MW, Praus M, Jung R, Hecking M, Metzig C, Gessner R. Ksp-cadherin is a functional cell–cell adhesion molecule related to LI-cadherin. Exp Cell Res. 2004;294(2):345–55. doi: 10.1016/j.yexcr.2003.11.022 . 15023525

[36] Wendeler MW, Jung R, Himmelbauer H, Gessner R. Unique gene structure and paralogy define the 7D-cadherin family. Cell Mol Life Sci. 2006;63(13):1564–73. doi: 10.1007/s00018-006-6014-x . 16791429 PMC11136315

[37] Eaton RC, Farley RD, Kimmel CB, Schabtach E. Functional development in the Mauthner cell system of embryos and larvae of the zebra fish. J Neurobiol. 1977;8(2):151–72. doi: 10.1002/neu.480080207 . 856948

[38] Takeichi M. The cadherin superfamily in neuronal connections and interactions. Nat Rev Neurosci. 2007;8(1):11–20. doi: 10.1038/nrn2043 . 17133224

[39] Thyme SB, Pieper LM, Li EH, Pandey S, Wang Y, Morris NS, et al. Phenotypic landscape of schizophrenia-associated genes defines candidates and their shared functions. Cell. 2019;177(2):478-491.e20. doi: 10.1016/j.cell.2019.01.048 . 30929901 PMC6494450

[40] Randlett O, Wee CL, Naumann EA, Nnaemeka O, Schoppik D, Fitzgerald JE, et al. Whole-brain activity mapping onto a zebrafish brain atlas. Nat Methods. 2015;12(11):1039–46. doi: 10.1038/nmeth.3581 . 26778924 PMC4710481

[41] Thomson RB, Igarashi P, Biemesderfer D, Kim R, Abu-Alfa A, Soleimani M, et al. Isolation and cDNA cloning of Ksp-cadherin, a novel kidney-specific member of the cadherin multigene family. J Biol Chem. 1995;270(29):17594–601. doi: 10.1074/jbc.270.29.17594 . 7615566

[42] Calì G, Zannini M, Rubini P, Tacchetti C, D’Andrea B, Affuso A, et al. Conditional inactivation of the E-cadherin gene in thyroid follicular cells affects gland development but does not impair junction formation. Endocrinology. 2007;148(6):2737–46. doi: 10.1210/en.2006-1344 . 17347311

[43] Pandey S, Shekhar K, Regev A, Schier AF. Comprehensive identification and spatial mapping of habenular neuronal types using single-cell RNA-seq. Curr Biol. 2018;28(7):1052-1065.e7. doi: 10.1016/j.cub.2018.02.040 . 29576475 PMC6042852

[44] Klingbeil K, Nguyen TQ, Fahrner A, Guthmann C, Wang H, Schoels M, et al. Corpuscles of stannius development requires FGF signaling. Dev Biol. 2022;481:160–71. doi: 10.1016/j.ydbio.2021.10.005 . 34666023

[45] Farrell JA, Wang Y, Riesenfeld SJ, Shekhar K, Regev A, Schier AF. Single-cell reconstruction of developmental trajectories during zebrafish embryogenesis. Science. 2018;360(6392):eaar3131. doi: 10.1126/science.aar3131 . 29700225 PMC6247916

[46] Sur A, Wang Y, Capar P, Margolin G, Prochaska MK, Farrell JA. Single-cell analysis of shared signatures and transcriptional diversity during zebrafish development. Dev Cell. 2023;58(24):3028-3047.e12. doi: 10.1016/j.devcel.2023.11.001 . 37995681 PMC11181902

[47] Jepsen MR, Kløverpris S, Mikkelsen JH, Pedersen JH, Füchtbauer E-M, Laursen LS, et al. Stanniocalcin-2 inhibits mammalian growth by proteolytic inhibition of the insulin-like growth factor axis. J Biol Chem. 2015;290(6):3430–9. doi: 10.1074/jbc.M114.611665 . 25533459 PMC4319012

[48] Kløverpris S, Mikkelsen JH, Pedersen JH, Jepsen MR, Laursen LS, Petersen SV, et al. Stanniocalcin-1 potently inhibits the proteolytic activity of the metalloproteinase pregnancy-associated plasma protein-A. J Biol Chem. 2015;290(36):21915–24. doi: 10.1074/jbc.M115.650143 . 26195635 PMC4571946

[49] Miller AH, Howe HB, Krause BM, Friedle SA, Banks MI, Perkins BD, et al. Pregnancy-associated plasma protein-aa regulates photoreceptor synaptic development to mediate visually guided behavior. J Neurosci. 2018;38(22):5220–36. doi: 10.1523/JNEUROSCI.0061-18.2018 . 29739870 PMC5977450

[50] Gjorcheska S, Paudel S, McLeod S, Paulding D, Snape L, Sosa KC, et al. Sox10 is required for systemic initiation of bone mineralization. Development. 2025;152(2):dev204357. doi: 10.1242/dev.204357 . 39791977 PMC11833171

[51] Kroll F, Powell GT, Ghosh M, Gestri G, Antinucci P, Hearn TJ, et al. A simple and effective F_0_ knockout method for rapid screening of behaviour and other complex phenotypes. Elife. 2021;10:e59683. doi: 10.7554/eLife.59683 . 33416493 PMC7793621

[52] Alassaf M, Daykin EC, Mathiaparanam J, Wolman MA. Pregnancy-associated plasma protein-aa supports hair cell survival by regulating mitochondrial function. Elife. 2019;8:e47061. doi: 10.7554/eLife.47061 . 31205004 PMC6594750

[53] Alassaf M, Halloran MC. Pregnancy-associated plasma protein-aa regulates endoplasmic reticulum-mitochondria associations. Elife. 2021;10:e59687. doi: 10.7554/eLife.59687 . 33759764 PMC8024009

[54] Hodorovich DR, Fryer Harris T, Burton DF, Neese KM, Bieler RA, Chudasama V, et al. Effects of 4 testing arena sizes and 11 types of embryo media on sensorimotor behaviors in wild-type and chd7 mutant zebrafish larvae. Zebrafish. 2024;21(1):1–14. doi: 10.1089/zeb.2023.0052 . 38301171 PMC10902501

[55] Kraus JM, Giovannone D, Rydzik R, Balsbaugh JL, Moss IL, Schwedler JL, et al. Notch signaling enhances bone regeneration in the zebrafish mandible. Development. 2022;149(5):dev199995. doi: 10.1242/dev.199995 . 35178545 PMC8959151

[56] Calì G, Gentile F, Mogavero S, Pallante P, Nitsch R, Ciancia G, et al. CDH16/Ksp-cadherin is expressed in the developing thyroid gland and is strongly down-regulated in thyroid carcinomas. Endocrinology. 2012;153(1):522–34. doi: 10.1210/en.2011-1572 . 22028439

[57] Yang X, Li Y, Liu G, Zha W, Liu Y. Cadherin-16 inhibits thyroid carcinoma cell proliferation and invasion. Oncol Lett. 2022;23(5):145. doi: 10.3892/ol.2022.13265 . 35350592 PMC8941525

[58] Peloggia J, Lush ME, Tsai Y-Y, Wood C, Piotrowski T. Environmental and molecular control of tissue-specific ionocyte differentiation in zebrafish. Development. 2024;151(20):dev202809. doi: 10.1242/dev.202809 . 39324331 PMC11528218

[59] Peloggia J, Münch D, Meneses-Giles P, Romero-Carvajal A, Lush ME, Lawson ND, et al. Adaptive cell invasion maintains lateral line organ homeostasis in response to environmental changes. Dev Cell. 2021;56(9):1296-1312.e7. doi: 10.1016/j.devcel.2021.03.027 . 33878346 PMC8142321

[60] Moccia M, Erro R, Nicolella E, Striano P, Striano S. Extreme startle and photomyoclonic response in severe hypocalcaemia. Epileptic Disord. 2014;16(1):84–7. doi: 10.1684/epd.2014.0638 . 24659607

[61] Green AJ, Wall AR, Weeks RD, Mattingly CJ, Marsden KC, Planchart A. Developmental cadmium exposure disrupts zebrafish vestibular calcium channels interfering with otolith formation and inner ear function. Neurotoxicology. 2023;96:129–39. doi: 10.1016/j.neuro.2023.04.006 . 37060951 PMC10518193

[62] Han P, Trinidad BJ, Shi J. Hypocalcemia-induced seizure: demystifying the calcium paradox. ASN Neuro. 2015;7(2):1759091415578050. doi: 10.1177/1759091415578050 . 25810356 PMC4374060

[63] Roper SN, Obenaus A, Dudek FE. Osmolality and nonsynaptic epileptiform bursts in rat CA1 and dentate gyrus. Ann Neurol. 1992;31(1):81–5. doi: 10.1002/ana.410310115 . 1543352

[64] Lu B, Zhang Q, Wang H, Wang Y, Nakayama M, Ren D. Extracellular calcium controls background current and neuronal excitability via an UNC79-UNC80-NALCN cation channel complex. Neuron. 2010;68(3):488–99. doi: 10.1016/j.neuron.2010.09.014 . 21040849 PMC2987630

[65] Vassilev PM, Ho-Pao CL, Kanazirska MP, Ye C, Hong K, Seidman CE, et al. Cao-sensing receptor (CaR)-mediated activation of K^+^ channels is blunted in CaR gene-deficient mouse neurons. Neuroreport. 1997;8(6):1411–6. doi: 10.1097/00001756-199704140-00018 . 9172145

[66] Pollak MR, Brown EM, Chou YH, Hebert SC, Marx SJ, Steinmann B, et al. Mutations in the human Ca^2+^-sensing receptor gene cause familial hypocalciuric hypercalcemia and neonatal severe hyperparathyroidism. Cell. 1993;75(7):1297–303. doi: 10.1016/0092-8674(93)90617-y . 7916660

[67] Zúñiga Mouret R, Greenbaum JP, Doll HM, Brody EM, Iacobucci EL, Roland NC, et al. The adaptor protein 2 (AP2) complex modulates habituation and behavioral selection across multiple pathways and time windows. iScience. 2024;27(4):109455. doi: 10.1016/j.isci.2024.109455 . 38550987 PMC10973200

[68] Jia S, Muto A, Orisme W, Henson HE, Parupalli C, Ju B, et al. Zebrafish Cacna1fa is required for cone photoreceptor function and synaptic ribbon formation. Hum Mol Genet. 2014;23(11):2981–94. doi: 10.1093/hmg/ddu009 . 24419318 PMC4014194

[69] Stearns G, Evangelista M, Fadool JM, Brockerhoff SE. A mutation in the cone-specific pde6 gene causes rapid cone photoreceptor degeneration in zebrafish. J Neurosci. 2007;27(50):13866–74. doi: 10.1523/JNEUROSCI.3136-07.2007 . 18077698 PMC6673616

[70] Bayley PR, Morgans CW. Rod bipolar cells and horizontal cells form displaced synaptic contacts with rods in the outer nuclear layer of the nob2 retina. J Comp Neurol. 2007;500(2):286–98. doi: 10.1002/cne.21188 . 17111373 PMC4238417

[71] Regus-Leidig H, Specht D, Tom Dieck S, Brandstätter JH. Stability of active zone components at the photoreceptor ribbon complex. Mol Vis. 2010;16:2690–700. . 21179232 PMC3002953

[72] Labun K, Montague TG, Gagnon JA, Thyme SB, Valen E. CHOPCHOP v2: a web tool for the next generation of CRISPR genome engineering. Nucleic Acids Res. 2016;44(W1):W272-6. doi: 10.1093/nar/gkw398 . 27185894 PMC4987937

[73] Thermes V, Grabher C, Ristoratore F, Bourrat F, Choulika A, Wittbrodt J, et al. I-SceI meganuclease mediates highly efficient transgenesis in fish. Mech Dev. 2002;118(1–2):91–8. doi: 10.1016/s0925-4773(02)00218-6 . 12351173

[74] Pujol-Martí J, Zecca A, Baudoin J-P, Faucherre A, Asakawa K, Kawakami K, et al. Neuronal birth order identifies a dimorphic sensorineural map. J Neurosci. 2012;32(9):2976–87. doi: 10.1523/JNEUROSCI.5157-11.2012 . 22378871 PMC6622018

[75] Lakhina V, Marcaccio CL, Shao X, Lush ME, Jain RA, Fujimoto E, et al. Netrin/DCC signaling guides olfactory sensory axons to their correct location in the olfactory bulb. J Neurosci. 2012;32(13):4440–56. doi: 10.1523/JNEUROSCI.4442-11.2012 . 22457493 PMC3356094

[76] Schultz J, Milpetz F, Bork P, Ponting CP. SMART, a simple modular architecture research tool: identification of signaling domains. Proc Natl Acad Sci U S A. 1998;95(11):5857–64. doi: 10.1073/pnas.95.11.5857 9600884 PMC34487

