# Supplementary figures and images for: Cadherin-16 regulates acoustic sensory gating in zebrafish through endocrine signaling
Source: PLoS Biol. 2025 May 2;23(5):e3003164. doi: 10.1371/journal.pbio.3003164 (PMC12077787; doi:10.1371/journal.pbio.3003164)

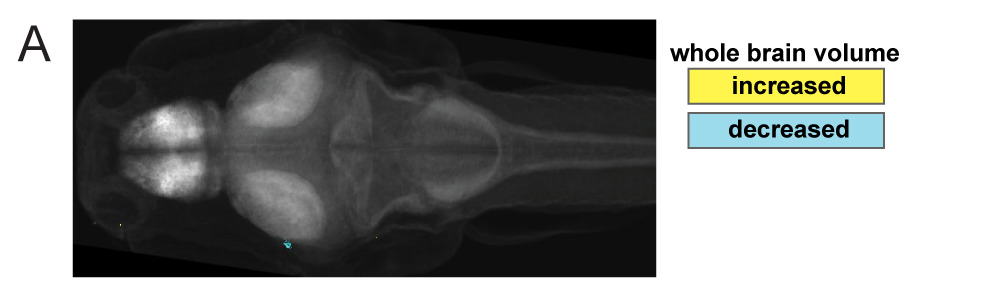

Supplement: S1 Fig — (A) Summary of whole-brain morphometric data for 6 dpf cdh16p173 mutants (n = 13) as compared to siblings (n = 16). Region-by-region differences in volume are indicated in yellow (regions that are larger in mutants) or cyan (regions that are smaller in mutants). Image is a summed stack of the significant delta medians of mutants over wild types. Note there are no colored pixels within the brain, indicating no significant differences between mutants and siblings across the annotated brain regions. (TIF) [file pbio.3003164.s001.tif]

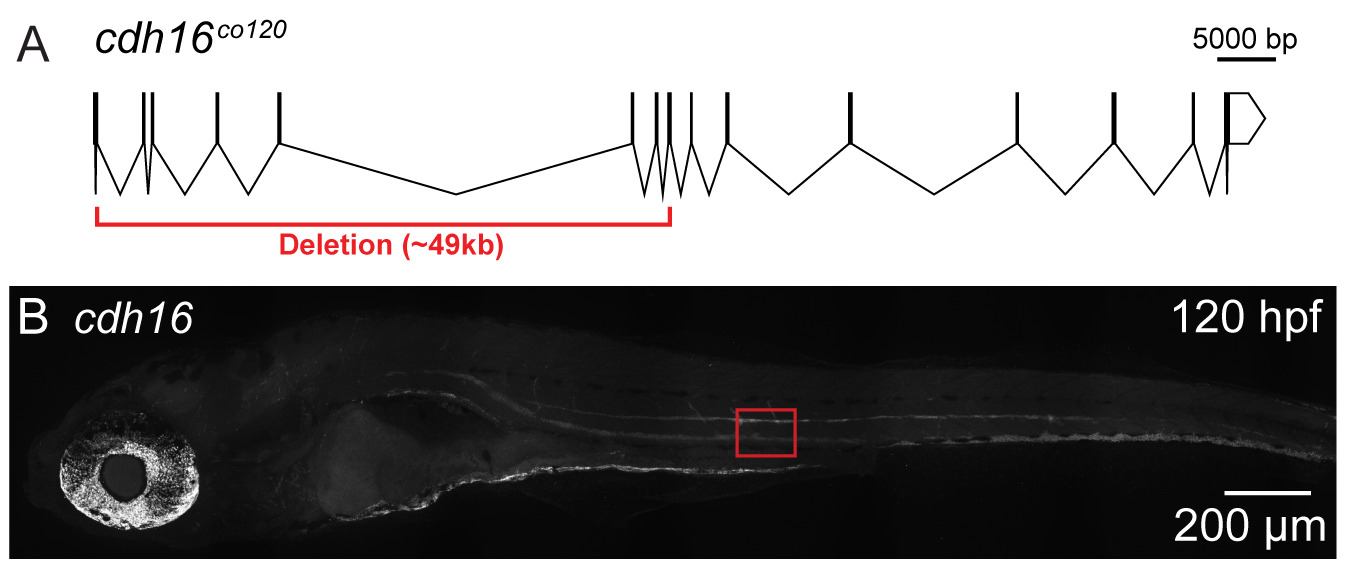

Supplement: S2 Fig — (A) Schematic of the large (approximately 49 kB) deletion allele that we generated in the cdh16 locus (cdh16co120). (B) in situ HCR image of cdh16co120 mutant larva using only probes that bind within the large deletion. The corpuscles of Stannius are not labeled. All other expression, including weak labeling in the head, can be considered background (representative image in Fig 4A). Shown is a representative image of cdh16co120; n = 4 larvae imaged. (TIF) [file pbio.3003164.s002.tif]

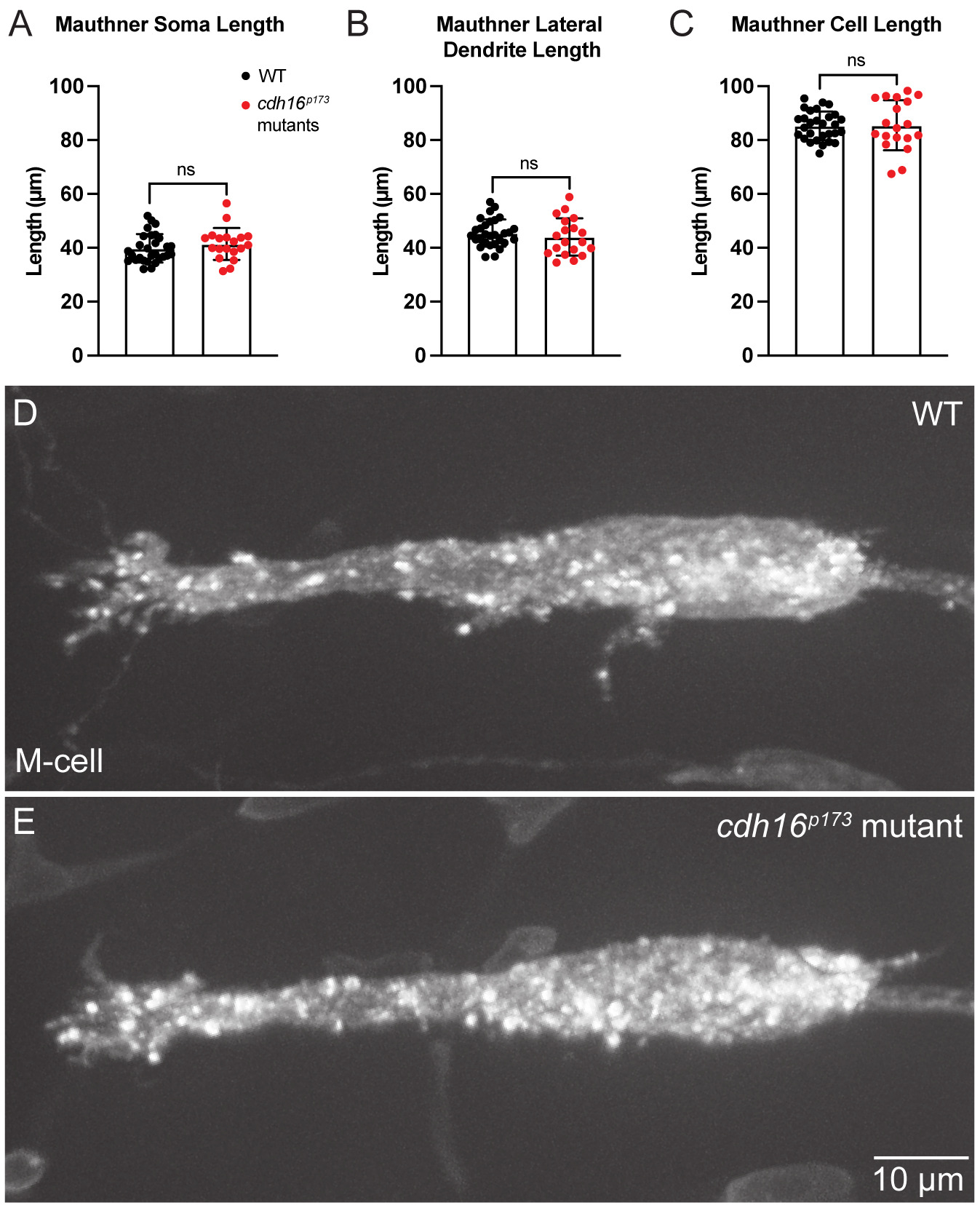

Supplement: S3 Fig — (A–E) Analysis of the Mauthner neurons responsible for the acoustic startle (SLC) response. (A–C) cdh16p173 mutants (n = 19) and wild type siblings (n = 29) have no differences between Mauthner soma length (A) p = 0.3169, lateral dendrite length (B) p = 0.4054, or total length (C) p = 0.9248, unpaired t test. Error bars represent SD. (D–E) No morphological differences are detected between the Mauthner cells of cdh16p173 mutant (bottom image) and WT larvae (top image). The data underlying this figure can be found in S1 Data. (TIF) [file pbio.3003164.s003.tif]

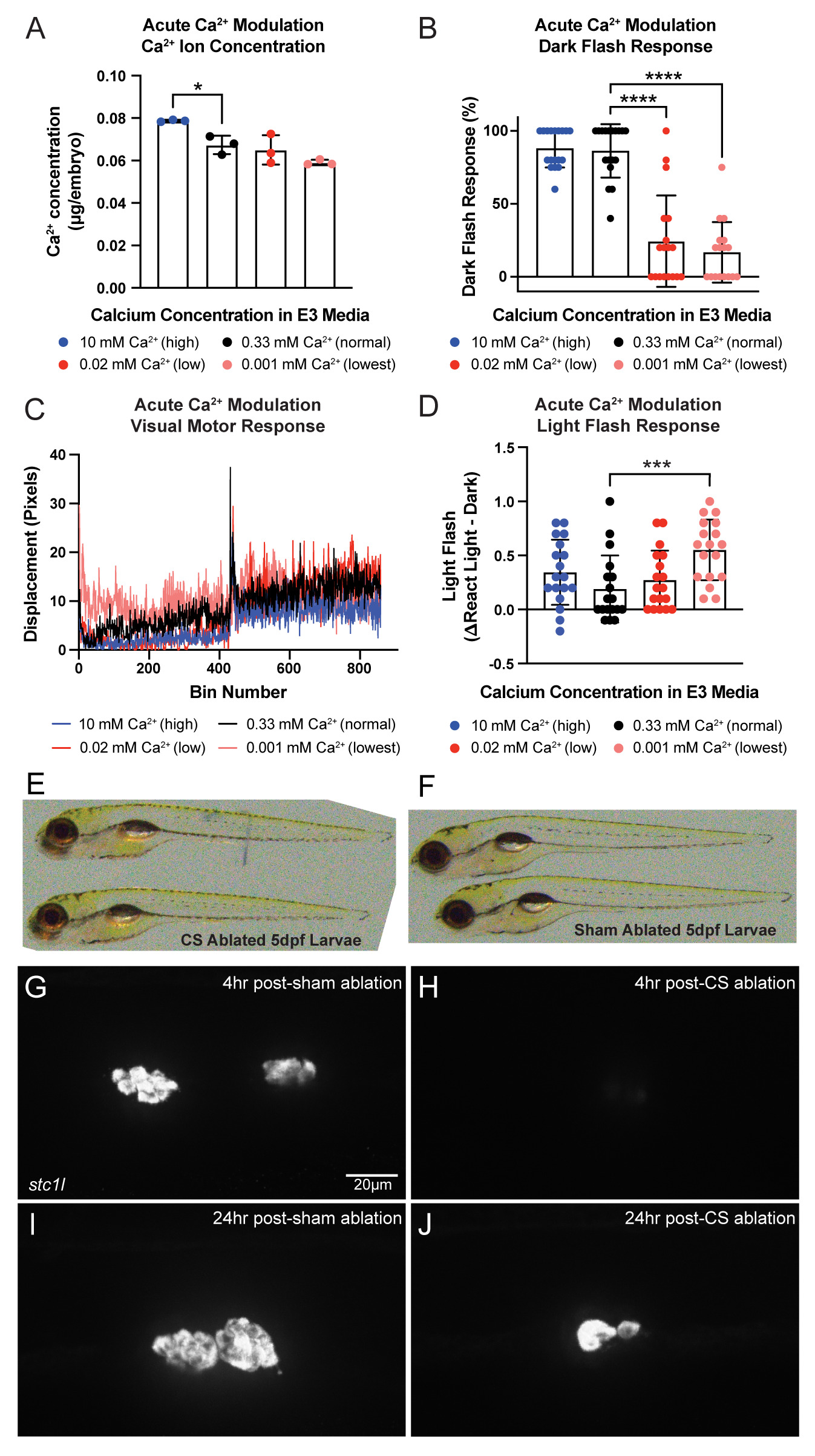

Supplement: S4 Fig — (A–D) E3 media with differing Ca2+ concentrations were applied to WT larvae at 5 dpf. Whole-body Ca2+ was measured 4 h later (A) or behavioral assays were performed 4 h later (B–D). (A) Exposure to high (10 mM) Ca2+ caused a slight but significant increase in whole-body Ca2+ levels, compared to normal 0.33 mM Ca2+ (*p = 0.024). Exposure to media containing low Ca2+ (0.02 and 0.001 mM) did not significantly affect whole-body Ca2+ levels (n = 3 biological replicates per condition, p = 0.8297, p = 0.093; one-way ANOVA with Dunnett’s multiple comparisons test). Error bars represent SD. (B) As is observed in pappaa mutant larvae, animals in 0.001 mM Ca2+ (n = 17) show decreased responding to dark flashes relative to siblings in 0.33 mM Ca2+ (n = 18) ****p < 0.0001, Kruskal–Wallis test with Dunn’s multiple comparisons test. Error bars represent SD. (C) Animals in the lowest (0.001 mM) Ca2+ concentration (n = 18) were more responsive to the lights-on stimulus in the visual motor assay as compared to their siblings in a normal 0.33 mM Ca2+ concentration (n = 18), p = 0.0033, Kruskal–Wallis test with Dunn’s test for multiple comparisons. Error bars represent SD. (D) Animals in 0.001 mM Ca2+ (n = 18) displayed more robust responses to a light flash than their siblings in 0.33 mM Ca2+ (n = 18) ***p = 0.0009, Kruskal–Wallis test with Dunn’s test for multiple comparisons. Error bars represent SD. (E–F) Images of 5 dpf WT larvae 24 h after either CS ablation (left) or sham ablation (right). Larvae with ablated corpuscles do not have visible pericardial edema. (G–J) stc1l HCR to visualize the CS after sham ablation (G, I) or CS ablation (H, J). Only a few stc1l-positive cells are present in the CS region 4 h after CS ablation (H), and stc1l expression is strongly reduced. By 24 h post-CS ablation, the structure has partially regenerated (J). Imaged n = 10 CS-ablated 4 h post-ablation, n = 5 sham-ablated 4 h post-ablation, n = 10 CS-ablated 24 h post-ablation, n = 5 sham-ablated 24 [file pbio.3003164.s004.tif]
